# Microbiome and plant relationship: a symbiosis against phytopathogens

**DOI:** 10.3389/fpls.2026.1722279

**Published:** 2026-03-04

**Authors:** Aizada Zholdasbek, Zhanar Tekebayeva, Kamshat Kulzhanova, Akhan Abzhalelov, Zhandarbek Bekshin, Dinara Yevneyeva, Merey Saylau, Xin Li, Zhouliang Tan, Zhaoqi Wang, Aslan Temirkhanov, Zhadyrassyn Nurbekova

**Affiliations:** 1 Department of Biotechnology and Microbiology, Lev Nikolaevich (LN) Gumilyov Eurasian National University, Astana, Kazakhstan; 2 Republican Collection of Microorganisms, Astana, Kazakhstan; 3 Chinese Academy of Science, Chengdu Institute of Biology, Chengdu, China

**Keywords:** antagonism, biological control, endophytes, microbiome, plant pathogen

## Abstract

Phytopathogens are among the major biotic stressors limiting global crop productivity. Conventional control methods, including chemical pesticides and fungicides, have contributed to pathogen resistance, environmental pollution, and soil degradation, highlighting the need for sustainable alternatives. This review highlights innovative, eco-friendly strategies that exploit plant–microbe interactions to enhance plant health and resilience across diverse agroecosystems. Rhizosphere-, phyllosphere-, and endosphere-associated microbial assemblages contribute to plant immune enhancement through induced systemic resistance, competitive nutrient exclusion, antimicrobial metabolite production, and mycoparasitism. The review emphasizes the functional roles of beneficial microbial communities and the emerging applications of synthetic consortia and bio-organic fertilizers to improving disease suppression, nutrient use efficiency, and soil fertility. In addition, recent progress in omics-based tools and microbial formulation technologies is discussed as a key driver for translating laboratory findings into practical field applications. However, large-scale implementation remains challenged by high research costs, limited metagenomic infrastructure, and the lack of standardized microbial formulations across environments. Strengthening institutional capacity, integrating omics-based tools, and improving technology transfer will be essential to unlock the full potential of microbiome-based pathogen control. Overall, this review highlights microbiome-based interventions as a sustainable alternative to chemical-intensive plant protection strategies under changing environmental conditions.

## 1 Introduction

Phytopathogens pose a serious threat to agriculture, causing significant annual crop losses of 20%–40%. The phytopathogens are the main causative agents of plant diseases, which include fungi, bacteria, viruses, and other pathogens that affect and reduce the economic value of important agricultural crops (Ayaz et al., 2023). The increase in population, coupled with growing food demand, has made a imperative to enhance crop yields. However, the effective environmental sustainability and economical affordability ways of controlling phytopathogens during production and post-production of agricultural crops remain a key task in achieving food security (Pandit et al., 2022).

Traditional methods of controlling pathogens, such as chemical fungicides, bactericides, and herbicides, ensure rapid and effective suppression of pathogens. However, their prolonged and uncontrolled use has environmental and social consequences (Akhtar et al., 2024). For example, organochlorine pesticides are very stable compounds; dichlorodiphenyltrichloroethane, in particular, can delay decomposition for 4 to 30 years depending on conditions (Sharma et al., 2019). Pesticides also exert selective pressure on target and non-target species, causing resistance in phytopathogens and microorganisms. This process is driven by two main mechanisms: joint selection and cross-resistance. These mechanisms have been reported to significantly complicate the control of resistant pathogens (Miller et al., 2022). The thriving of the activities of phytopathogens has been linked to extreme weather events such as rising temperatures and increased humidity, contributing to the spread of the phytopathogens and frequent disease outbreaks. For instance, thermophilic bacteria such as *Burkholderia glumae* and *Ralstonia solanacearum* as well as other viruses actively replicate in response to high temperatures (Saeed et al., 2021). These challenges have necessitated adapting existing plant protection strategies and developing new control methods. Therefore, maintaining healthy soil conditions, such as optimal aeration and organic matter content, is critical for establishing an active and diverse microbiome (Islam et al., 2020).

A prominent approach to be considered in replacing the traditional method of controlling phytopathogens is the utilization of diverse plant microbiome communities of microorganisms, such as bacteria, fungi, viruses, and archaea that exist in different relationships with plants (Glick and Gamalero, 2021). Scientific studies have demonstrated that plant microbiomes support plant health, enhance nutrient uptake, and promote growth while also contributing to plant resilience against biotic and abiotic stresses (Flemer et al., 2022; Pradhan et al., 2022; Enebe and Babalola, 2019). Inoculating plants with beneficial bacterial or fungal strains can mitigate the effects of phytopathogens and influence specific plant traits. These are being explored to improve plant productivity and stress tolerance through microbiome engineering (Azarbad and Junker, 2024).

To suppress pathogenic pressure and improve plant resilience, consortia of microorganisms that mimic natural ecological interactions are assembled to create synthetic microbial communities. Yin et al. (2022) expressed that seven synthetic microbial communities (SynComs) demonstrated protective properties against *Rhizoctonia solani* AG8 infection. Additionally, some SynComs tend to increase plant fresh root mass compared with other individual strains in response to environmental stress. This indicates a potential positive effect on plant growth. However, upon this positive effect of SynComs, their effectiveness in controlling phytopathogens did not exceed that of individual bacteria, indicating the need for further investigation of their interactions (Chen et al., 2019). Therefore, the objective of this review is to assess the establishment and effects of microbial inoculants and microbial communities on plants, particularly focusing on their ability to enhance plant resilience and reduce the severity of plant diseases under different ecological conditions (Darbon et al., 2024). This review emphasizes microbiome structures and their interaction with plants, mechanisms of microbiome-mediated pathogen suppression and microbiome application in pathogen control, while also discussing major challenges and future perspectives of microbiome-based disease management strategies.

## 2 Plant symbiosis with microorganisms and host–microorganism interaction

There are three known types of symbiosis based on Tikhonovich’s and Provorov’s (2003) classification: facultative, ecologically obligate, and genetically obligate. Facultative symbiotic microbes can interact with the host using the same genes and/or functions during adaptation in the external environment; among them are rhizosphere bacteria, asymptomatic, ambivalent endophytes, and epiphytes. Ecologically obligate symbionts interact with the host through genes and that function in the external environment. Examples of these symbionts are *Rhizobium*, nitrogen-fixing endophytes, most phytopathogenic fungi, and bacteria. For genetically obligate microbes, the host is the only possible habitat. Genetically, obligates are glomus fungi, mycoplasmas, or viruses (Tikhonovich and Provorov, 2003).

### 2.1 Beneficial plant-associated microorganisms

Plant-associated microbiota form functionally organized communities in the rhizo-, phyllo-, and endosphere, enhancing plant resilience and productivity under both biotic and abiotic stresses. The effects of these communities depend on the host genotype, microbial community structure, and environmental conditions, as the same taxa may exhibit either mutualistic or opportunistic traits (Rolfe et al., 2019; Santoyo, 2022). Within these interactions, arbuscular mycorrhizal fungi play a key role by initiating symbiosis through strigolactone-induced root colonization and arbuscle formation (Besserer et al., 2006; Maclean et al., 2017). This results in mycorrhiza-induced resistance, mediated by both direct activation of plant defense pathways and indirect mycorrhizospheric effects, including the restructuring of bacterial communities and the suppression of phytopathogens (Cameron et al., 2013).

Alongside mycorrhizal associations, dark septate endophytes colonize plant roots and form melanized hyphae and microsclerotia that compete with pathogens for resources and space and exhibit high functional plasticity (Jumpponen et al., 1998; Mandyam and Jumpponen, 2005, 2015; Kageyama et al., 2008; Li et al., 2018; Santos et al., 2021). Dark septate endophytes have also been reported to modify the composition of bacterial and fungal communities in soil by increasing the proportion of beneficial taxa and reducing pathogen abundance (Chao et al., 2021; Netherway et al, 2024). Among fungal symbionts, *Trichoderma* species represent one of the most widely used model systems, as they combine the ability to colonize the root surface with strong antagonistic activity against other fungi (Vinale et al., 2008).

### 2.2 Microbial interactions in the plant–soil system

Microbial interactions within the plant–soil system encompass a wide range of ecological strategies, including mutualism, commensalism, competition, antagonism, and lysis, which collectively shape the structure and functional activity of soil microbial communities. These interaction types are mediated by diverse molecular mechanisms (**Table 1**), including metabolite exchange, secretion of signaling molecules, and production of antimicrobial compounds, ultimately influencing nutrient availability and ecosystem stability.

**Table 1:** Major microbial interactions and their associated molecular mechanisms in the plant–soil system.

Interaction types	Description	Molecular mechanisms	Microbial groups	Reference
Mutualism	Mutual benefit for all participants in the interaction	Transport of carbon compounds and organic acids	Rhizobia-legumes	Tharanath et al. (2024)
Commensalism	One microorganism benefits without having a noticeable effect on the other	Hydrolysis of *B. cereus* peptidoglycan with subsequent assimilation of its fragments by CF bacteria	*Bacillus cereus (Firmicutes)* and *Cytophaga–Flavobacterium* group bacteria	Peterson et al. (2006)
Competition	Competition between interacting microorganisms for energy sources and nutrients	Secreting specific hydrolytic enzymes Produce bacteriocins, antifungal compounds, and antibiotics Siderophore production	Bacteria and fungi	Wang and Kuzyakov (2024)
Antagonism (microbe–microbe)	Suppression of the growth or vital activity of competing microorganisms	Antibiotic/antimicrobial compound production	Antagonistic bacteria/fungi	Moënne-Loccoz et al. (2014)
Lysis	Destruction of cells of other microorganisms	Production of lytic enzymes	Endophytic bacteria *Bacillus* spp.	Bodhankar et al. (2017)

The interactions between hosts and microorganisms and their ecological roles are not fixed. These can vary for many reasons. Most microorganisms can change their relationship with the host at different life stages, mostly as a response to changes in the environment such as pH, moisture, temperature, and nutrients. Microorganisms can change their relationship with the host from pathogenic to symbiotic and/or from mutualism to parasitism. This plasticity reveals the potential toward protective mutualism under agricultural management.

## 3 The structure of microbiomes and their interaction with plants

**Figure 1:**
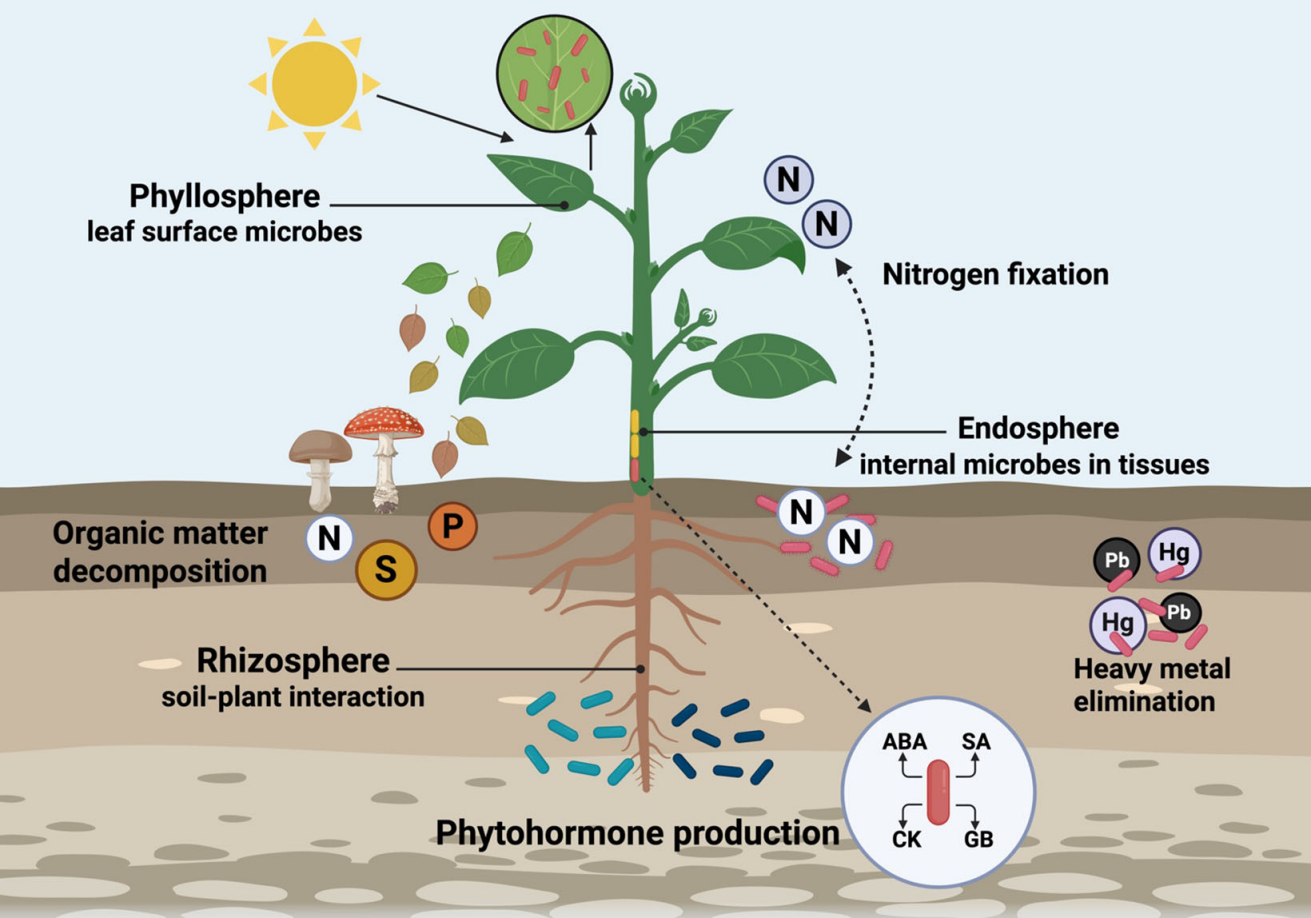
This diagram shows the main ecosystems of microbes involved in symbiosis with plants and their distinct plant-associated zones. Created using BioRender.com.

Plant-associated microbial assemblages occupy distinct ecological compartments of the plant system (Dastogeer et al., 2020). These include the rhizosphere, which is the region surrounding the roots, the phyllosphere referring to the surface of the leaves, and the endosphere (inside of plant tissues). The microbial communities perform a critical ecological function and establish specific ecosystems by colonizing soil, roots, leaves, and internal tissues of plants (**Figure 1**).

Recent plant microbiome research increasingly relies on integrated meta-omics approaches that combine metagenomics with metatranscriptomics, metaproteomics, and metabolomics to link microbial community composition with expressed functions and metabolite-mediated interactions (Arıkan and Muth, 2023; Kimotho and Maina, 2024). This integration enables identification of active functional guilds involved in nutrient cycling, pathogen suppression, and plant immune modulation, which cannot be inferred from taxonomic data alone. Moreover, coupling metagenomics with metabolomics allows tracking of microbiome functional shifts under biotic stress and supports rational selection of strains for synthetic community design and in-planta functional validation (Copeland et al., 2025).

### 3.1 Rhizosphere and microbial activities

The rhizosphere encompasses the area surrounding plant roots where root exudates are secreted, containing a variety of organic compounds such as sugars, amino acids, organic acids, and secondary metabolites. The root exudates create a unique environment that attracts and stimulates microbial activity (Jacoby et al., 2020). This enhances nutrient uptake and fosters feedback mechanisms between plants and soil that provide protection against pathogens (Sun et al., 2021). Despite the well-known role of secondary metabolites, there is a lack of practically oriented research that addresses the understanding and importance of the interactions between of specific metabolites and microbes.

These interactions are essential to the rhizosphere microbiota, which comprises diverse bacterial and fungal communities that contribute to plant nutrition, protection, and growth (Mendes et al., 2013). One of the most frequently studied microorganisms of the rhizosphere is mycorrhizal fungi, which form a symbiosis with plant roots, accessing otherwise unavailable nutrients and satisfying up to 80% of the plant nutrient requirements (Dastogeer et al., 2020). For instance, nitrogen-fixing bacteria such as *Rhizobia* are known for forming root nodules on legumes, where they convert atmospheric nitrogen into bioavailable forms (Ledermann et al., 2021). However, not all rhizobacterial strains exhibit the same effectiveness in root colonization and interaction with different plant species. As a result, insufficient root colonization is one of the main reasons for the variability in biological control effectiveness. The development and adaptation of the microbiota association with plant roots enhance soil fertility and reduce reliance on chemical fertilizers. This reduces the risks associated with the use of chemical and other inorganic fertilizers (Saeed et al., 2021).

Additionally, bacteria like *Azospirillum brasilense* synthesize phytohormones indolyl-3-acetic acid (auxin), which promote root elongation, branching (Compant et al., 2019), nutrient acquisition, and plant resistance to adverse environmental conditions. Other genera, such as *Pseudomonas* and *Bacillus*, produce growth-promoting substances and biocontrol agents. For instance, the *Bacillus subtilis* strain JN005 effectively suppressed the development of rice pyricularia, reducing the incidence by 76%-79% and increasing yield to 524.4 g/m^2^, while stimulating plant growth and activating protective enzymes (Zhu et al., 2022).

In addition, phosphate-mobilizing bacteria actively interact in the rhizosphere, facilitating the mobilization of insoluble phosphorus into bioavailable forms, a function particularly important in nutrient-deficient soils where phosphorus availability is limited (Elhaissoufi et al., 2021).

It is important to note that the health of the rhizosphere health depends on soil physicochemical properties, environmental conditions, and plant genotypes, which collectively influence the composition and activity of the microbiome (Pantigoso et al., 2022). Plant genotype and the process of plant domestication have been shown to influence microbiome composition. Agroecosystem studies prove that plant species and subspecies exert a stronger influence on microbial communities than geographical location or fertilization methods (Xiong et al., 2021).

### 3.2 Phyllosphere

The phyllosphere involves the aerial surfaces of plant leaves and stems, hosting a diverse but comparatively less abundant microbiota than the rhizosphere, and this is due to nutrient limitations. Climatic conditions, such as changes in temperature, humidity, and precipitation, can influence ecological processes that shape the microbial community of the phyllosphere, for instance, by altering the physicochemical properties of leaves (Huang et al., 2023). Drought has been reported to alter the microbiome of wild rice in both aboveground and belowground plant parts. In leaves and stems, drought reduces microbial community diversity and stability, whereas in roots and the rhizosphere, no significant changes are observed. Belowground microbial networks remain resilient due to strong interactions between drought-sensitive taxa. Additionally, drought enriches belowground microbial communities with *Actinobacteria* but does not affect their abundance in aboveground plant parts (Xie et al., 2021).

The most common groups found in the phyllosphere are *Pseudomonas* spp., *Firmicutes*, *Acidobacteria*, *Cyanobacteria*, *Bacteroidetes*, and *Actinobacteria* (Legein et al., 2020). Wax-like materials and organic molecules that are mostly secreted from the leaf’s surfaces influence the microbiome composition by selectively encouraging or discouraging the specific microbial taxa. For instance, the organic molecules contained in these secretions are a key factor in the adaptation of microorganisms to nutrient-poor conditions. Thus, *Methylobacterium* sp. and *Sphingomonas* sp. demonstrate high adaptability due to their ability to metabolize a wide range of carbon sources, which gives them a competitive advantage in nutrient-deficient conditions (Santos and Olivares, 2021). In addition, plant growth-promoting bacteria in the phyllosphere significantly enhance plant productivity by synthesizing natural growth regulators. For example, foliar applications of isolates such as *Methylobacterium arborescens, Bacillus subtilis, Stenotrophomonas maltophilia*, and *Enterobacter hormaechei* on maize increased shoot biomass and nutrient uptake. The underlying mechanisms included the production of indole-3-acetic acid and nitrogenase activity (Abadi et al., 2020).

Moreover, certain bacterial consortia, such as *Pantoea, Pseudomonas*, and *Actinobacteria* spp., reduce infections caused by pathogens like *Pseudomonas syringae* pv. *glycinea*, improving photosynthetic efficiency and optimizing water use efficiency, which is crucial for stress tolerance and crop yield improvement (Agbavor et al., 2022).

At the same time, the phyllosphere microbiome promotes bioremediation through the ability of some bacteria to degrade pollutants. An example is the strain *Flavobacterium* sp. YD4 isolated from the phyllosphere of rapeseed. It uses dichlorvos, chlorpyrifos, and foxime as the only sources of phosphorus, demonstrating effectiveness in decomposing pesticides. Despite promising insights, the phyllosphere remains an uninvestigated microbial section compared with the endosphere and rhizosphere. The gap persists in how various stressors affect microbial functionality and interactions with host immunity. By fulfilling these gaps, the advancements of phyllosphere-based biocontrol, foliar inoculant development, and bioremediation strategies would be covered. This study shows that epiphytic bacteria can be a promising tool for *in situ* bioremediation, especially in conditions unsuitable for soil or aquatic bacteria (Ning et al., 2012).

### 3.3 Endosphere

The endospheric microbiome directly penetrates plant tissues, eliciting distinct responses from the host. Unlike external zones, endosphere diversity is lower and influenced by plant organ specificity (Liu et al., 2012). Endophytic microbial assemblages colonize internal plant tissues and form specialized symbiotic associations mediated by chemotaxis, host surface attachment, and tissue invasion processes. Endophytes are transmitted either horizontally, usually between plants, or vertically (across generations). Vertical transmission often provides greater benefits to the plant (Gupta et al., 2021). The plant endosphere is a highly specialized environment where different bacterial species can thrive depending on exudates, nutrient conditions, and microbial specificity. The composition of endophytic bacterial communities varies depending on the plant organ they colonize, soil type, climate, management practices, and biotic and abiotic stressors (Babalola and Adedayo, 2023). The roots of *P. cupana* exhibited the highest richness and diversity of endophytic bacteria, whereas leaves and seeds contained fewer species. This supports the hypothesis that endophytic communities adapt to the specific anatomical and physiological conditions of each plant organ (Liotti et al., 2018). Beneficial endophytic bacteria, including representatives of *Enterobacter, Pseudomonas, Burkholderia, Bacillus*, and *Azospirillum*, have been identified in various plant species and act as biocontrol agents (Vandana et al., 2021). In an effort to establish a stable symbiosis, endophytes produce numerous compounds that promote host plant growth and enhance environmental adaptation (Fadiji and Babalola, 2020). For instance, *Streptomyces* spp. not only produce siderophores and solubilize phosphate but are also known to produce a variety of enzymes, including amylase, chitinase, cellulase, invertase, lipase, keratinase, peroxidase, pectinase, protease, phytase, and xylanase, which convert complex nutrients into simpler mineral forms (Jog et al., 2016). Endophytic microbiomes have shown great potential in promoting plant growth, yet major gaps remain in understanding their transmission strategies the lack of understanding of the organ-specific interactions. Closing these gaps is essential to harnessing endophytes in agriculture, especially under climate-stress conditions.

### 3.4 Ecological and molecular integration of plant microbiome niches

Despite spatial separation, the plant microbiome is established as a functionally interconnected system that integrates the rhizosphere, endosphere, and phyllosphere. This integration is maintained by systemic plant signals, including phytohormones and mobile metabolites, which coordinate microbial activity across spatially distinct compartments (Wankhade et al., 2025). The rhizosphere plays a central role in the assembly and stability of the plant microbiome. Rhizosphere microorganisms contribute to the relative stability by participating in plant nutrient acquisition and serving as the primary source of microbial recruitment for both the endo- and phyllosphere. In turn, epiphytic and endophytic microbes associated with aboveground organs promote plant growth and regulate local and systemic immune responses (Akter et al., 2025).

**Figure 2:**
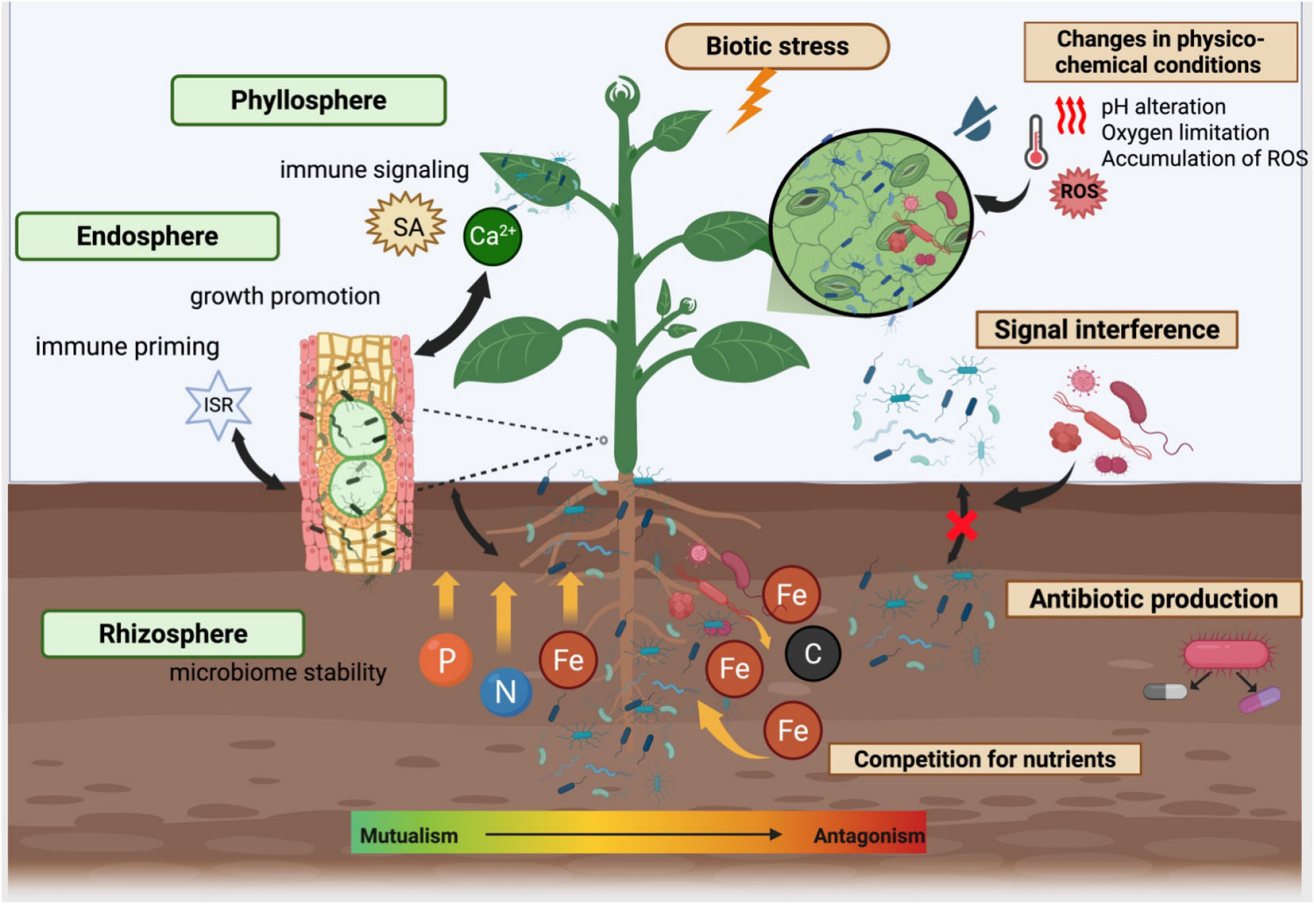
Ecological and molecular crosstalk among rhizosphere, endosphere, and phyllosphere microbiomes regulating plant–microbe interactions under biotic stress. Created using BioRender.com.

Microorganisms that exhibit protective properties under moderate stress conditions may become neutral or even detrimental when plant immune responses intensify or when resources become limited. This shift reflects a transition from cooperative modes of coexistence toward competitive and suppressive interactions aimed at maintaining overall system survival and is strongly influenced by resource availability, the physiological state of the host plant, and microbial community density (**Figure 2**).

Competition for carbon, nitrogen, micronutrients, and adhesion niches is a key factor shaping microbial communities in the rhizosphere, endosphere, and phyllosphere (Jones et al., 2018). Root exudates, comprising sugars, amino acids, organic acids, and secondary metabolites, establish resource gradients that selectively stimulate the growth of specific microbial taxa (Canarini et al., 2019). Under stress conditions, these gradients can both enhance microbial community resistance and promote rapid compositional shifts in response to changes in plant physiological status. It has been demonstrated that the outcome of microbiome–pathogen interactions is often determined by competition for iron. Specifically, bacteria-producing siderophores inaccessible to the pathogen *Ralstonia solanacearum* suppress its growth and reduce disease severity, whereas “compatible” siderophores may, conversely, facilitate infection (Gu et al., 2020).

Plants actively modulate the composition of root exudates in response to biotic and abiotic cues. Colonization by beneficial microorganisms is associated with a shift toward exudate profiles that support mutualistic interactions, whereas pathogen pressure can induce the secretion of antimicrobial compounds (Badri et al., 2013). For example, *Ralstonia solanacearum* infection increases the release of caffeic acid in tomato roots, leading to reduced soil bacterial diversity and altered microbiota composition. Caffeic acid partially suppresses pathogen growth while increasing the relative abundance of Proteobacteria and Actinobacteria and reducing Firmicutes, Acidobacteria, and Verrucomicrobia (Gu et al., 2016).

Additional ecological gradients arise from localized hypoxia within plant tissues. During infection by necrotrophic fungi such as *Botrytis cinerea* and *Alternaria brassicicola*, oxygen concentrations in highly restricted leaf regions can drop below 1%. This phenomenon results from increased respiration of infected tissues, competition for oxygen between the plant and the pathogen, and reduced O_2_ diffusion in necrotic zones (Loreti and Perata, 2020). Alterations in redox status create unfavorable conditions for aerobic mutualists, promoting a shift toward microaerophilic or anaerobic microorganisms and leading to rapid microbiome restructuring at infection sites.

Phyllosphere microorganisms are sensitive to systemic signals originating from the root zone, including hormonal and metabolic changes induced by infection. In particular, root colonization by arbuscular mycorrhizal fungi (AMF) markedly reshapes the phyllosphere microbiome of alfalfa during pathogen attack, resulting in increases of ACE and Chao1 diversity indices by 25.38% and 20.14%, respectively, alongside statistically significant changes in community structure. Concurrently, AMF enhance plant defense responses (TI, PPO, SA), indicating coordinated regulation of plant immunity and the leaf-associated microbial community (Wang et al., 2025). Under strong defense signaling, plants may restrict nutrient availability or activate mechanisms that suppress even previously beneficial symbionts. Some endophytes, such as bacteria carrying the *nahG* gene, can degrade salicylic acid (SA), thereby preventing excessive immune activation. However, under intense SA signaling, as shown for *Epichloë* fungal endophytes, endophyte-derived alkaloid production is reduced (Benjamin et al., 2022).

Functional shifts in plant-associated microbial communities are frequently mediated by signal interference, whereby degradation of quorum-sensing (QS) signals disrupts the coordinated expression of genes involved in pathogenicity, biofilm formation, and secondary metabolite biosynthesis. Quorum sensing has been shown to regulate secondary metabolite production in dominant microbial taxa, thereby influencing community structure while the overall taxonomic composition of the microbiome remains relatively stable (Armes et al., 2025). However, the ecological consequences of QS disruption are context-dependent. AHL-mediated regulation underlies the biocontrol activity of many beneficial bacteria, and nonspecific degradation of AHL signals in such systems can reduce their effectiveness by weakening competitive advantages and antagonistic activity against phytopathogens (Molina et al., 2003).

In general, the plant microbiome should be viewed not as a collection of spatially isolated communities, but as a dynamically integrated system linking the rhizosphere, endosphere, and phyllosphere at both functional and signaling levels. Plant resistance to pathogens and the efficiency of symbiotic interactions emerge from multilevel coordination between the host plant and its associated microbiome. Understanding the ecological and molecular principles that connect microbial plant niches provides a theoretical foundation for developing targeted microbiome management strategies to enhance plant resilience and optimize biocontrol in agroecosystems.

## 4 Mechanisms of microbiome-mediated pathogen suppression

The plant immune system and microbial community are closely interconnected, creating a complex system of protection against pathogens and adverse environmental conditions. Unlike animals, plants are living sessile lifestyle; thus, they have developed unique defense mechanisms, including physiological and chemical reactions. **Table 2** illustrates the mechanisms of action and protection of various microbial communities. One of these mechanisms is the recognition of pathogens using special receptors that detect molecules associated with pathogens (pathogen-associated molecular patterns, PAMPs). This interaction triggers PAMP-triggered immunity (PTI), which involves the synthesis of antimicrobial proteins and the production of reactive oxygen species (Lü et al., 2022). The adaptation of these mechanisms to improve plant resilience to pathogens will enhance plant performance and productivity and contribute to the realization of achieving food security.

**Table 2:** The mechanism of action and protection of various microbial communities.

Microbiome	Host plant	Pathogen	Microorganisms	Mechanisms	References
Phyllosphere	Citrus	*Diaporthe citri*	*Pantoea, Methylobacterium Sphingomonas*	Antimicrobial metabolites secretion, iron competition	Li et al. (2022)
	Grape	*Uncinula necator, Botrytis cinerea*	*Bacillus subtilis*	Antibiotics and cell wall hydrolase production	Maachia et al. (2015)
	Hair er shen	*Fusarium oxysporum*	*Pseudomonas* spp.	Direct antagonism and indirect disruption of the pathogen virulence factor biosynthesis	Yuan et al. (2024)
	Rice	*Bipolaris oryzae, Sarocladium oryzae*	*Pseudomonas aeruginosa, Bacillus* spp.	Production of volatile, non-volatile organic compounds, fluorescent pigments and hydrogen cyanide (HCN), protease enzymes	Sobanbabu et al. (2024)
	Tomato	*Pseudomonas syringae, Alternaria solani*	*Rhizobium* spp., *Bacillus subtilis*	Production of protease, cellulase activation of salicylic acid (SA)- dependent immune response	Shao et al. (2023)
Endosphere	Oilseed rape	*Leptosphaeria biglobosa, Sclerotinia sclerotiorum, Phoma lingam, Verticillium dahlae*	*Pseudomonas, Enterobacter, Serratia, Stenotrophomona, Bacillus, Staphylococcus*	Phosphorus solubilization, siderophore production, induced resistance pathways (SA, JA) and antimicrobial production	Schmidt et al. (2021)
	Banana	*Fusarium oxysporum*	*Enterobacter* spp. *Kosakonia* spp. *Klebsiella* spp.	Production of ACC deaminase	Liu et al. (2019)
	Canola	*Xanthomonas campestris, Sclerotinia sclerotiorum, Leptosphaeria maculans*	*Pseudomonas viridiflava*	Induction of host resistance mediated by SA and JA signaling, production of antimicrobial compounds	Romero et al. (2019)
	Tobacco	*Ralstonia solanacearum*	*Burkholderia*	Antagonism	Tao et al. (2024)
	Grape	*Botrytis cinerea, Colletotrichum gloeosporioides, Phytophtora infestans*	*Bacillus velezensis*	Production of antifungal substances	Hamaoka et al. (2021)
Rhizosphere	Citrus	Dry root rot pathogens	*Bacillus* spp., *Stenotrophomonas* spp., *Sphingobacterium* spp.	Production of hydrolytic enzymes, hydrogen cyanide, indole-3-acetic acid, antimicrobial compounds	Ezrari et al. (2021)
	Indian mustard	*Alternaria brassicae*	Rhizobacteria isolates: HMR25, HMR48, HMR70, HMM44, HMM89, HMR32, HMR 33, WHA64	Production of indole-3-acetic acid, HCN, siderophore	Sharma et al. (2018)
	Tea	Root rot pathogens	*Serratia marcescens*	Production of hydrolytic enzymes and plant growth promoting metabolites	Dhar Purkayastha et al. (2018)
	Tomato	*Tomato yellow leaf curl virus (TYLCV)*	*Bacillus amyloliquefaciens*	Enhanced expression of resistance-related genes and activity of defense enzymes	Guo et al. (2019)
	Tomato	*Ralstonia solanacearum*	*Bacillus velezensis, Pseudomonas fluorescens*	Antagonism, competition for space and nutrients	Elsayed et al. (2020)

### 4.1 Plant systemic resistance

One of the most notable mechanisms of microbiome-mediated suppression is induced systemic resistance. The beneficial microbiomes prime the entire plant system for enhanced defense against a wide range of phytopathogens and pests (Ali et al., 2023). This process does not act as a direct counteraction against pathogens, relying more on plant-growth-promoting bacteria. The beneficial microorganisms like plant-growth-promoting bacteria, secrete elicitors such as siderophores, lipopolysaccharides, lipopeptides, and volatile organic compounds, which help trigger plant immune responses (Meena et al., 2020). Microorganisms closely interact with the plant root system, initiating a cascade of molecular signals that spread throughout the plant. These signals lead to the activation of multiple defense pathways, including the synthesis of phytohormones such as salicylic acid, jasmonic acid, and ethylene, as well as the upregulation of resistance-related genes (Kour et al., 2024). For instance, surfactin produced by *Bacillus subtilis* triggers both salicylic and jasmonic acid-dependent pathways, enhancing wheat resistance against *Zymoseptoria tritici* (Le Mire et al., 2018).

This dual activation of the salicylic acid and jasmonic acid pathways is critical because of their generally antagonistic nature. Salicylic acid signaling is primarily associated with defense responses against biotrophic pathogens, organisms that feed on living host tissue. Jasmonic acids, which often go with ethylene, govern defense against necrotrophic pathogens, which kill host cells, as well as herbivorous insects. Previous studies have shown that this cross-activation allows plants to calibrate their immune responses according to the nature of the threat and the environmental context. For instance, the research done by Shameer and Prasad (2018) highlights how *B. subtilis* and other plant-growth-promoting bacteria act as inducers of systemic resistance. Similarly, the study by Song et al. (2023) supports the idea that the dual activation of the salicylic acid and jasmonic acid pathways provides a robust and adaptable immune system, especially under combined abiotic and biotic conditions.

Moreover, fungal filtrations, such as those from *Penicillium* spp., can induce lignification at pathogen penetration sites and increase reactive oxygen species levels, contributing to induced systemic resistance (fungal-mediated ISR). These adaptations underline the importance of microbial interactions in preemptively enhancing plant defenses (Koike et al., 2001). Research by Yuan et al. (2018) reveals that aboveground pathogen infections alter root exudation patterns, reshaping rhizospheric microbial communities to enhance induced systemic resistance. It is assumed that when plants are infected, the system alarm changes in root exudates. This contributes to the formation of stable microbiomes and hereditary resistance in the plant future (Yuan et al., 2018). Certain microbial species, including *Pseudomonas, Bacillus*, and mycorrhizal fungi, are effective inducers of induced systemic resistance (Bakker et al., 2013).

**Figure 3:**
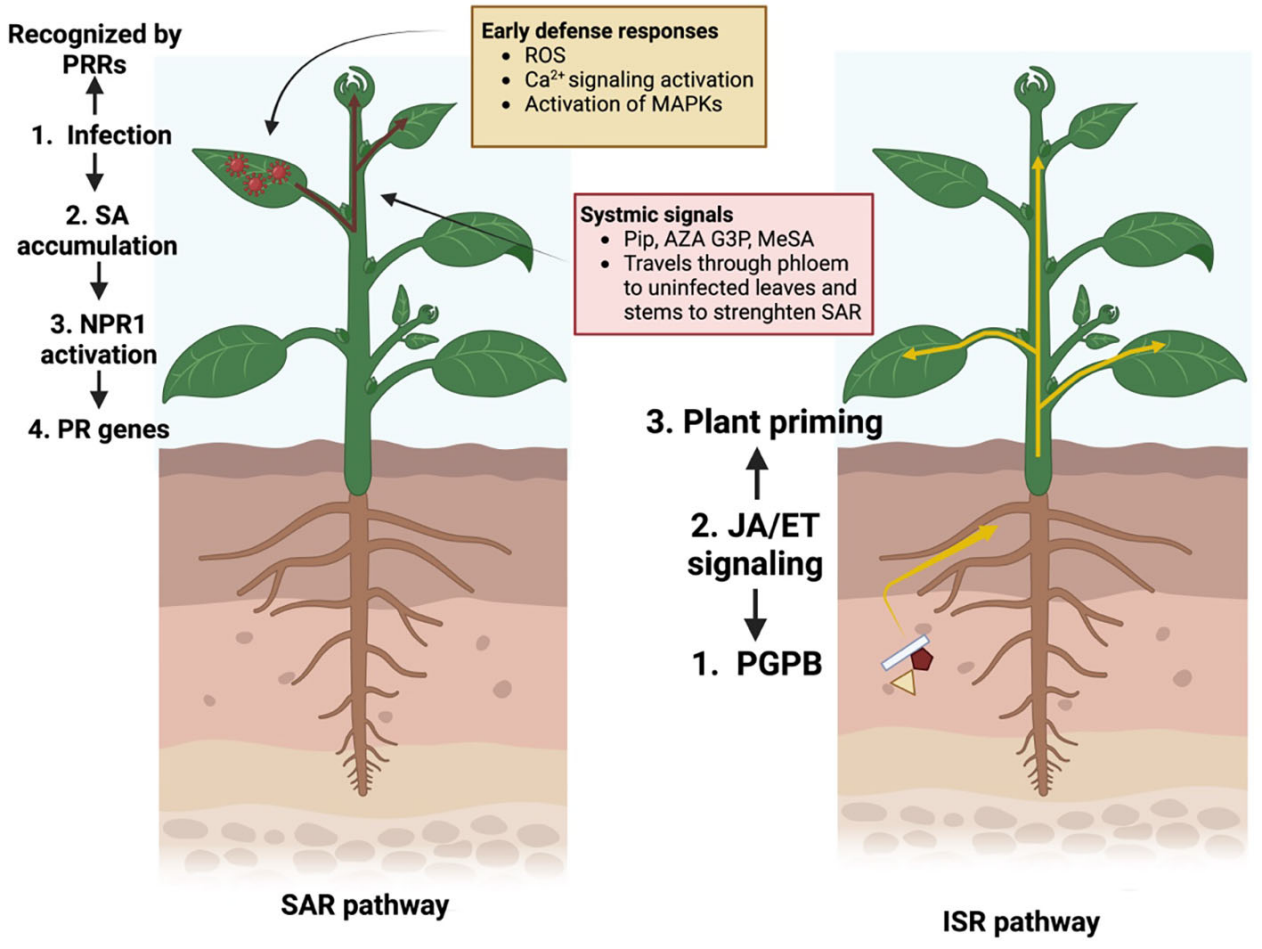
Systemic acquired resistance (SAR) and induced systemic resistance (ISR) pathways mechanisms. Created using BioRender.com.

In contrast to induced systemic resistance, systemic acquired resistance (SAR) is activated upon pathogen presence and reduces the likelihood of disease development upon subsequent pathogen encounters (**Figure 3**). For example, *Bacillus subtilis* produces elicitors, including acetoin, which induce SAR through salicylic acid- and NPR1-dependent pathways (Zehra et al., 2021). In addition, elicitors activate various defense mechanisms, including hypersensitive responses, H_2_O_2_ accumulation, and PR-protein synthesis, which enhance resistance to pathogens such as *Tobacco Mosaic Virus, Phytophthora infestans*, and *Botrytis cinerea*. For example, reticin A has been shown to effectively induce SAR against *Tobacco Mosaic Virus* by increasing the expression of key resistance genes (Wang et al., 2021). Li et al. (2022) demonstrated that the bacterial strain *Priestia megaterium* JR48 not only induced plant resistance to *Xanthomonas campestris* pv. *campestris* (Xcc) but also activated key defense mechanisms, including hydrogen peroxide (H_2_O_2_) accumulation, callose deposition, and the upregulation of defense-related genes. Additionally, JR48 promoted lignin biosynthesis and increased free salicylic acid (SA) as well as the expression of pathogenesis-related genes.

An increased transcript or activity levels of pathogenesis-related genes (e.g., PR3 encoding chitinase), lipoxygenases (LOX), and antioxidant enzymes (peroxidases, glutathione-S-transferases) are measurable molecular markers of microbiome-induced priming. These markers offer practical assays for induced systemic resistance induced by both bacteria and fungi.

### 4.2 Competition for nutrients

In soil environments rich in diverse microorganisms and nutrients, microbial communities engage in intense competition for resources. The competition is mediated through various antagonistic mechanisms, including mycoparasitism, the production of inhibitory compounds, and the exclusion of pathogens from access to key nutrients (Prajapati et al., 2020). This is an indirect mechanism of microbial antagonism, in which the suppression of phytopathogens occurs due to the more efficient development of limited environmental resources, rather than due to their direct damage. Yeasts are frequently used in competitive interactions due to their rapid reproduction in nutrient-rich environments, particularly in the presence of sugars and root exudates. In the study by Gross et al., several yeast species were assessed for their biocontrol potential. The results demonstrated that the competitiveness and antagonistic activity of *Candida subhashii* were significantly enhanced when N-acetylglucosamine served as the sole carbon source. Moreover, *M. pulcherrima* and *A. pullulans* were effective in suppressing diseases phyllosphere diseases (Gross et al., 2018). In addition, yeast does not produce toxic metabolites, which makes them biologically safe agents for plant protection (Kowalska et al., 2022). *Sphingomonas* spp. inhabiting the phyllosphere of Arabidopsis *thaliana* has been shown to suppress the growth of *Pseudomonas syringae* pv. tomato by competing for available carbon sources. The protective strains utilized sugars present on the leaf surface. The metabolic niche overlap index between antagonistic strains and the pathogen is 0.43 and ranged from 0.26 to 0.30 for non-protective strains. These findings indicate that the ability to utilize the same carbon substrates as the pathogen allows commensal bacteria to occupy similar ecological niches and limit pathogen proliferation through direct competition for nutrients (Innerebner et al., 2011).

In microbial antagonism, competition for nutrients plays a key role in determining the effectiveness of pathogen suppression. For example, strain *K. apiculata* 34–9 inhibits the growth of *Penicillium italicum* through nutrient competition. However, in the presence of sufficient nutrients, *P. italicum* can still develop despite the presence of the antagonist. Studies have shown that *K. apiculata* utilizes a limited range of carbon sources, whereas *P. italicum* can metabolize a much broader spectrum. This difference in nutritional preferences determines their interaction dynamics and may reduce biocontrol effectiveness (Liu et al., 2013). This highlights the key feature of competition—its pronounced dependence on environmental conditions.

Certain microorganisms also compete for iron by producing siderophores—small, high-affinity iron-chelating compounds that sequester iron from the environment, making it accessible only to organisms possessing specific siderophore receptors and thereby limiting availability to competitors (Deb and Tatung, 2024). Pseudomonas fluorescens spp. is an effective competitor for ferric iron (Fe^3+^), producing two types of siderophores—pyoverdine (a fluorescent pigment) and pyochelin (a non-fluorescent pigment) (Schalk, 2008). Results indicate that suppression of *Ralstonia solanacearum* growth by *Pseudomonas* spp. consortia occurred primarily through iron competition mediated by siderophore production. Under iron-deficient conditions (Fe ≈ 6 μM), an inhibitory effect on the pathogen was observed in 73.81% of bacterial consortia, whereas under high iron availability (Fe = 50 μM), this effect was noted in only 61.9% of cases. In greenhouse trials, application of the consortia reduced the incidence of bacterial wilt in tomato plants from 66.7% (control) to 57.7%, confirming the efficacy of iron-dependent competition in plant protection (Shao et al., 2024).

### 4.3 Antimicrobial compound production

Microorganisms with natural disease resistance have significant potential for antibiotic production (Du et al., 2025). Endophytic fungi have attracted considerable research interest in this field due to their remarkable ability to produce novel bioactive compounds, many of which exhibit broad-spectrum antimicrobial activity (Caruso et al., 2022).

The strain *Pseudomonas palleroniana* has been found to suppress the growth and sporulation of key pathogens (*Fusarium oxysporum, Botrytis cinerea*, etc.) by releasing volatile organic compounds (e.g., dimethyl disulfide, squalene). This strain also inhibits the synthesis of virulence factors and enriches beneficial microbiota, thereby enhancing plant resistance and reducing disease incidence (Yuan et al., 2024). Microbiological interactions also extend to the dynamics between bacteria and fungi, as seen in the antagonism between *Pseudomonas piscium* from the wheat spikelet microbiome and the phytopathogenic fungus *Fusarium graminearum*. *P. piscium* produces phenazine-1-carboxamide, which disrupts histone acetylation in fungi, leading to reduced fungal growth, virulence, and mycotoxin biosynthesis. This finding demonstrates that certain bacterial strains can manipulate fungal epigenetics, offering new strategies for disease control (Chen et al., 2018). In another study, the strain *Pseudomonas fuscovaginae* UPB0736 was sequenced, revealing a novel cyclic lipopeptide named asplenin, which plays a role in antifungal activity and swarming motility. Additionally, syringotoxin was confirmed to exhibit strong antifungal activity against *Rhizoctonia solani*. Studies on mutant strains showed that fuscopeptin and syringotoxin play a crucial role in rice sheath lesions, affecting the number of panicles produced (Ferrarini et al., 2022).

Members of the *Bacillus* species produce various bioactive compounds—including cyclic lipopeptides and other peptides. During the logarithmic growth stage, members of *Bacillus* spp. produce various bioactive compounds, including cyclic lipopeptides (iturins, fengycins, surfactins) and other peptides, and more. These antibiotics are known to inhibit the growth of pathogens by disrupting the synthesis of microbes’ cell walls (Khadieva et al., 2018). The *Streptomyces* spp. also produce antibiotics, for instance, tetracycline and streptomycin, which disrupt protein synthesis in bacteria by binding the 30s ribosomal subunit. However, microbial consortia often outperform monocultures (of the same *Bacillus* or *Streptomyces*) due to the synergistic pathogen-suppression mechanisms. For example, *Bacillus subtilis* can secrete metabolites that stimulate *Streptomyces* to produce antibiotics on a large scale (Lahlali et al., 2022; Lelyak and Lelyak, 2013; Tyagi et al., 2024; Jaiswal et al., 2022).

Dark septate endophytes produce melanin and other secondary metabolites that act directly on the pathogen. Secondary metabolites reduce oxidative damage and increase fungal survival in plants. Under iron-limited soil conditions, dark septate endophyte-derived antibiotics and siderophores can inhibit various pathogens, such as *Rhizoctonia solani* (Caruso et al., 2022).

### 4.4 Mycoparasitism

Mycoparasitism is a specialized form of antagonism in which one fungus parasitizes another, suppressing its vital activity. Mycoparasitism involves obligatory physical contact between the antagonistic microorganism and the fungal pathogen. As natural inhabitants of the rhizosphere, *Trichoderma* species serve as prominent biocontrol agents due to their mycoparasitic capabilities. They can recognize fungal pathogens, direct growth to them, secrete enzymes that destroy the cell wall, wrap around their hyphae, and decompose them, thereby suppressing the development of the disease in plants (Guzmán-Guzmán et al., 2025). In a study on sugarcane, *Trichoderma harzianum* strain T20 exhibited antagonistic activity against the red rot pathogen *Colletotrichum falcatum*. The strain showed high mycoparasitic potential by producing an increased number of cell wall-degrading enzymes, especially chitinases and β-1,3-glucanases (Elamathi et al., 2018). Thereby, *Sphaerodes mycoparasitica* SMCD 2220–01 effectively reduced the production of the major *Fusarium* mycotoxins under co-cultivation. Specifically, it decreases levels of zearalenone by 97%, deoxynivalenol by 89%, 3-ADONE by 58%, and 15-ADONE by 72%. The analysis revealed the formation of less toxic sulfate metabolites, indicating the strain’s capacity for mycotoxin biodetoxification (Kim and Vujanovic, 2017).

Unlike the necrotrophic mechanism, *Sphaerodes mycoparasitica* employs biotrophic mycoparasitism, relying on ABC transporter genes to eliminate toxins and form infectious structures, thereby allowing it to effectively suppress the development of *Fusarium graminearum* in the absence of beneficial plant-associated microbiota (Kim and Vujanovic, 2022). Similarly, *Clonostachys chloroleuca* (strain 67-1) is active against *Sclerotinia sclerotiorum*, the causal agent of white mold. Transcriptomic analysis revealed a key transcription factor gene, crtf, whose expression increased by more than 20-fold within 8 h after contact with pathogen sclerotia. Deletion of crtf genes reduces mycoparasitic activity, whereas its restoration restores biocontrol properties (Beye et al., 2018). In natural microbiomes, mycoparasitism rarely functions in isolation but acts synergistically with other antagonistic mechanisms. An example of such a complex action is demonstrated in the study of *Pythium oligandrum*, which effectively suppresses *Aphanomyces euteiches*, a root pathogen in legumes, through a combination of direct mycoparasitism, activation of over 1,000 plant immune-related genes, accumulation of antifungal isoflavonoids, and secretion of elicitor proteins POD1 and POD2 that trigger hormonal defense pathways (Kim and Vujanovic, 2022).

The findings indicate that mycoparasites employ a wide range of antagonistic strategies, from direct enzymatic attack on pathogen structures to molecular regulation and adaptation during biotrophic interactions. Further investigation into these mechanisms offers opportunities to develop highly effective biofungicides and sustainable agricultural technologies.

In natural microbial communities, competition for nutrients, antibiosis, and mycoparasitism rarely acts in isolation and they are frequently expressed concurrently. Despite their co-occurrence, these mechanisms are fundamentally distinct. Competition represents an indirect mode of antagonism driven by ecological niche overlap and limitation of pathogen access to essential environmental resources. Antibiosis, by contrast, is a direct chemical mechanism of inhibition mediated by the production of antimicrobial metabolites that suppress pathogen growth, development, or virulence, independent of nutrient availability. Mycoparasitism occupies a unique position as a specialized form of direct trophic interaction, requiring physical contact between the antagonist and the pathogen and involving enzymatic degradation of pathogen structures or biotrophic suppression.

## 5 Application of microbiomes in pathogen control

In the face of increasing pressure on agriculture due to diseases and pests, the use of microbiome-based strategies offers a sustainable alternative to chemical remedies. These methods are not only effective but also environmentally safe, making them increasingly attractive to farmers and agronomists seeking sustainable farming practices. Therefore, harnessing natural symbiotic relationships between plants and microorganisms provides a significant advantage over chemical pesticides (Du et al., 2025; Foo et al., 2017).

Inoculation with beneficial microbial strains has a positive impact on plants’ resistance to pathogens in various ways. Research indicates that inoculating leaf-associated microbiota can effectively protect tomatoes from the pathogen *Pseudomonas syringae*, with a degree of protection depending on the inoculum dose. The protective effect was observed to reduce pathogen growth at low microbial doses, primarily through competitive exclusion and microbial antagonism, and to involve plant immune activation (Berg and Koskella, 2018). Additionally, the transplantation of entire fungal microbial communities, as demonstrated in the case of *E. koolauensis*, shows that microbial diversity can significantly enhance plant resistance to pathogens. The microbiome, especially when transplanted into *E. koolauensis* leaves, consisted of a diverse range of fungal microorganisms (mycoflora) naturally occurring in plants of the *Myrtaceae* family. Certain fungal species within this community exhibited antagonistic behavior toward pathogens, such as hyperparasitism and competitive exclusion (Chock et al., 2021).

Zhong Wei et al. examined the application of bio-organic fertilizers enriched with *Bacillus* spp. for controlling of bacterial wilt of tomatoes caused by *Ralstonia solanacearum*. The group reported that the use of bio-organic fertilizers significantly reduced disease incidence during the spring season. This maintains *R. solanacearum* populations in the soil below 5.8 log CFU/g prior to transplanting, contributing to more effective biocontrol. The application of these fertilizers was shown to activate plant defense pathways, enhance soil microbial diversity, and stimulate the activity of beneficial microorganisms. Furthermore, *Bacillus* strains were capable of degrading exopolysaccharides, which are critical for pathogen biofilm formation, thus reducing the virulence of *R. solanacearum* (Wei et al., 2011). These results highlight the dual role of bio-organic fertilizers in pathogen suppression and soil health improvement.

Another approach uses the natural microbiota associated with seeds, particularly *Pantoea* spp., to protect tomatoes from *Pseudomonas syringae* pv. tomato. The study demonstrated that the natural microbiota of tomato seeds effectively reduced disease severity, with consistent effects across different tomato cultivars. The optimal dose of protective bacteria was 40 CFU per seed, corresponding to the naturally occurring density on seeds. Increasing the bacterial dose did not reduce pathogen load but did lead to a further reduction in disease symptoms. UV-treated (non-viable) bacteria did not alleviate disease symptoms; however, certain strains significantly reduced pathogen density (p < 0.05), likely by stimulating of the plant’s immune response (Morella et al., 2019).

Furthermore, bacteriophages offer a unique tool for genetic manipulation within microbial communities. As natural DNA carriers, phages can be used to selectively remove specific pathogenic strains or introduce beneficial genes that promote plant growth (Ke et al., 2021). One study demonstrated that increasing phage application frequency improved control of *Ralstonia solanacearum* density, resulting in a significant reduction in disease incidence. Repeated phage treatments reduced bacterial wilt incidence up to 84% in field conditions and 67% in greenhouse experiments, accompanied by a four-log reduction in pathogen density. In parallel, phage application increased microbiome diversity and selectively enriched Actinobacteria, particularly *Streptomyces* and *Nocardioides*, which exhibited strong antagonistic activity against the pathogen. The combined use of phages and these bacteria enhanced disease control efficacy up to 79%, indicating a synergistic effect (Wang et al., 2024).

The application of the microbiome in pathogen control (**Table 3**) illustrates the diverse and innovative ways microbiome-based strategies can be leveraged to address agricultural challenges. By fostering beneficial microbial communities and harnessing their natural capacities, researchers and farmers can develop sustainable and effective solutions to protect crops, promote plant health, and reduce reliance on chemical interventions.

**Table 3:** The application of different types of microbiomes.

Application	Description	Example use cases	Mode of action	References
Inoculation of soil with microbial strains	Direct addition of beneficial bacteria/fungi into soil to colonize the rhizosphere or suppress pathogens.	*Trichoderma*, *Bacillus subtilis* for root rot	Mycoparasitism, competition, antibiosis, ISR	Elamathi et al. (2018); Ezrari et al. (2021)
Transplantation of fungal communities	Transfer of native or adapted beneficial fungal consortia into new soil.	Arbuscular mycorrhizal fungi in degraded soils	Mycoparasitism, ISR, competition	Chock et al. (2021); Shao et al. (2024); Smith and Read (2002)
Organic fertilizers + bacteria	Enrichment of composts/manure with plant-beneficial microbes.	Compost tea + *Azotobacter* or *Pseudomonas*	Competition, antibiosis, ISR	Compant et al. (2019); Liu et al. (2013); Miller et al. (2022)
Microbiota + seeds	Coating seeds with bacteria/fungi to protect from early pathogens and support growth.	Seed coating with *Bacillus amyloliquefaciens*	Competition, antibiosis, ISR	Ke et al. (2021); Lü et al. (2022); Pantigoso et al. (2022)
Microbial community cocktails	Use of mixtures of microbes with complementary functions and modes of action.	Commercial bioinoculants (BioGro, Rizotech)	All modes—depend on microbial diversity in the mix	Dastogeer et al. (2020); Ledru et al. (2022); Liu et al. (2020); Li et al. (2021); Niu et al. (2017); Xie et al. (2021)
DSE inoculation	Soil or seed *Phialocephala* spp. for treatment with wheat or wilt suppression.	Melanized root *Glycyrrhiza urakensis* endophytes field trial	ISR, siderophore-mediated iron competition.	Caruso et al. (2022); Schmidt et al. (2021)

### 5.1 Synthetic microbial communities as a microbiome engineering tool

Advances in microbiome research have led to the development of synthetic microbial communities that enable the investigation of microbial interactions and their regulation for improving the efficacy of microbial inoculants. These communities simplify the complexity of natural microbiomes into experimental “modules,” enabling analysis of community composition, strain ratios, colonization dynamics, plant growth, immune activation, and disease suppression. The applicability of this approach has been demonstrated in multiple models and applied studies. For example, synthetic communities comprising four to seven bacterial strains significantly reduced *Fusarium* infection in plants a combining direct pathogen suppression with induction of host systemic resistance (Li et al., 2021; Niu et al., 2017). Under field conditions, consortia of indigenous bacteria have likewise been shown to enhance plant resistance to pathogen complexes, indicating the relevance of opportunistic interactions between plants and their microbiomes (Santhanam et al., 2015).

The principles of SynCom design include bottom-up and top-down design (Kong et al., 2018). The bottom-up design starts with isolating and culturing individual strains, followed by testing their combinations for synergism, stability, and functional redundancy. However, *in vitro* traits often poorly predict behavior within complex communities; therefore, functional and ecological screening is required. In contrast, the top-down design aims to reduce the complexity of natural microbial communities associated with a target phenotype (Shayanthan et al., 2022; Zhang et al., 2025; Lyu et al., 2024).

In the development of both approaches, it is essential to consider the functional complementarity of community members, enabling activity against different pathogen life forms and infection stages. However, the realization of these functions is only possible when inter-microbial interactions are well coordinated, as pronounced antagonism within a synthetic microbiome can destabilize the community and lead to the loss of key functional strains. In turn, community stability is largely determined by the niche competence of its members—their ability to efficiently and persistently colonize the target compartment (rhizosphere, endosphere, or phyllosphere). In practice, this is achieved by selecting strains originating from the same compartment in which the SynCom will be applied (Shayanthan et al., 2022).

The integration of omics approaches accelerates the development of microbial consortia and enables the rational selection of strains for synthetic communities. During the selection stage, these approaches facilitate a shift from mere taxonomic presence to functional relevance by identifying microorganisms that actively express genes and synthesize metabolites associated with pathogen suppression and plant stress tolerance (Arıkan and Muth, 2023; Kimotho and Maina, 2024). The combined use of metagenomic and metabolomic data supports the identification of functional microbial groups, the selection of strains for SynCom assembly, and the monitoring of target inoculant functions in plants and soils (Copeland et al., 2025).

However, the interpretation of meta-omics data remains constrained by incomplete metabolite annotation, difficulties in linking functions to specific taxa within complex communities, and the high sensitivity of microbial activity to variable environmental conditions (Arıkan and Muth, 2023; Copeland et al., 2025). When integrated with SynCom approaches, meta-omics renders consortium development more hypothesis-driven, progressing from community profiling and functional selection to cultivation (Jing et al., 2024; Marín et al., 2021), multilevel strain characterization, and experimental validation of stability and functionality (Kong et al., 2018; Russ et al., 2023; Xu et al., 2025).

Reviews of SynCom studies demonstrate that effective consortia are not formed by simply aggregating “beneficial” strains, but rather by combining taxa with diverse ecological strategies, which enhances community-level stability and protective effects. SynCom experiments also uncover interactions that remain undetectable in single-strain analyses, underscoring that plant protection is often determined by the maintenance of balance within microbiome (Duran et al., 2018; Kong et al., 2018; Shayanthan et al., 2022).

Overall, microbiome-based biocontrol strategies, including synthetic microbial consortia, are considered promising tools for sustainable agriculture; however, their efficacy and reproducibility under field conditions remain major scientific and practical challenges (Kong et al., 2018). Controlled *in vitro* systems primarily reflect direct antagonism of individual strains but fail to capture the complexity of natural microbial communities, the micro niche structure of soils, and the influence of the host plant (Compant et al., 2019). High spatial and physicochemical heterogeneity of soils, fluctuations in temperature and humidity (Copeland et al., 2025; Tariq et al., 2025), and competition with resident microbiota and priority effects often hinder the establishment of introduced strains (Fadiji and Babalola, 2020). As a result, strains exhibiting strong *in vitro* activity may lose efficacy within complex communities, whereas moderately active microorganisms can display high in planta performance due to interactions with the host plant and the rhizosphere (Besset-Manzoni et al., 2019). Therefore, the successful field application of SynComs requires assessing colonization capacity (Tanaka et al., 2025), stability under specific soil and climatic conditions, and plant species-dependent effects. Modern approaches emphasize stress tolerance, optimized formulation, and standardized delivery methods (e.g., seed coating), which collectively enhance the reproducibility and reliability of biocontrol in real agroecosystems (Arnault et al., 2024; Shayanthan et al., 2022).

## 6 Challenges and future perspective

The study of interactions between plants and microbes opens new opportunities for developing sustainable, environmentally friendly farming methods. However, significant challenges exist on the path to effectively harnessing plant microbiomes for crop protection. To improve clarity, the major limitations can be categorized into technical barriers, ecological constraints, and policy and biosafety considerations.

At the same time, recent advances in microbiome engineering, synthetic community design, and multi-omics integration represent emerging research frontiers that offer novel opportunities to translate mechanistic insights into field-applicable solutions. Identifying how these emerging approaches can be scaled and stabilized under real agronomic conditions remains a key future research priority.

### 6.1 Technical barriers (omics tools and reproducibility)

Advanced omics technologies, including metagenomics, metabolomics, and next-generation sequencing, are essential for characterizing microbial diversity and functional traits; however, these methods require expensive equipment, skilled personnel, and standardized analytical pipelines, which limit reproducibility across laboratories and regions. Additionally, large-scale microbiome research and practical implementation are often constrained by high costs and limited modern laboratory infrastructure. Advanced techniques such as metagenomics, metabolomics, and next-generation sequencing require expensive equipment, skilled personnel, and well-equipped facilities, which are often unavailable in developing or resource-limited regions. The absence of standardized tools and analytical platforms further restricts the ability to isolate, characterize, and test microbial strains efficiently, slowing progress toward field-level applications. An emerging and novel research direction is the development of low-cost, portable sequencing platforms, cloud-based bioinformatics pipelines, and machine-learning-assisted functional prediction tools that can reduce dependency on highly specialized infrastructure. However, future studies must focus on benchmarking these tools against conventional platforms and validating their predictive accuracy under diverse environmental conditions.

Strengthening institutional research capacity and promoting technology transfer are therefore essential to overcome these technical and economic barriers. Recent studies emphasize the need for intelligent reprogramming strategies and improved bioinoculant formulations to enhance reproducibility and functional performance under controlled and field conditions (Jabran et al., 2023, 2024).

Future research should also prioritize the development of real-time functional monitoring approaches, such as biosensor-based detection of microbial activity and metabolite fluxes in planta, to verify whether introduced microbiomes express target traits after field application.

### 6.2 Ecological limitations (host specificity and microbiome stability)

The complexity and variability of plant microbiomes have remained a primary challenge, as each plant species harbors a unique assemblage of microbes. The unique assemblage of microbes depends on numerous factors, such as soil composition, climate, and even the plants’ chemical profile. This complexity hinders the development of universal plant protection strategies, since the behavior of the microbiome can vary considerably with changing environmental conditions (Zahoor et al., 2024). The rhizosphere is characterized by continuous competition between beneficial and pathogenic microorganisms, further complicating the maintenance of an optimal microbial balance. Recent advances in synthetic microbial communities (SynComs) represent a novel approach to reduce ecological unpredictability by assembling functionally complementary microbial consortia rather than relying on single-strain inoculants. However, future research must determine how SynComs evolve over time under field conditions and whether functional stability can be maintained across multiple cropping seasons. Another serious concern is the potential for pathogenic microorganisms to develop resistance. The results depend on pest populations, treatment methods, and environmental conditions, which require optimization of composition, dosage, etc. Rather than directly combating pathogens, the proposed strategy involves reinforcing protective microbiomes through the application of organic fertilizers, microbial inoculation, and crop rotation. Studies indicate that only a small fraction of microbial taxa is associated with plant genotypes and that there is a significant interaction between genotype and environment, suggesting that plants may select microbes that are most beneficial under specific conditions.

Host specificity and limited persistence of introduced strains reduce long-term microbiome stability, making repeated applications necessary and reducing cost-effectiveness, particularly under fluctuating climatic conditions.

Future studies should focus on identifying plant genetic traits that promote recruitment of beneficial microbiota, enabling microbiome-assisted breeding strategies. Integrating plant genomics with microbiome selection represents a promising but underexplored frontier for sustainable disease management.

### 6.3 Policy, regulatory, and biosafety aspects

Beyond scientific challenges, regulatory frameworks and biosafety assessments remain major barriers to large-scale deployment of microbiome-based products. Microbial inoculants must undergo rigorous evaluation to assess environmental persistence, risks of horizontal gene transfer, and potential impacts on native microbial communities.

A novel challenge emerging with next-generation bioinoculants is the regulation of multi-strain consortia and genetically enhanced microbes, for which existing regulatory systems are often inadequate. Future policy development should incorporate microbiome-specific risk assessment models that consider community-level behavior rather than individual strains.

The application of microbiomes in agricultural systems is already extending beyond theoretical research, and this is being actively implemented in practice. Biological fertilizers and growth stimulants, biopesticides, enhancement of soil microbiomes through plant genetic modification, and the reduction of methane emissions in livestock all demonstrate the potential of microbiomes to enhance the resilience of agroecosystems and mitigate environmental impacts. However, inconsistent regulatory policies across countries and limited guidelines for multi-strain microbial consortia delay commercialization and farmer adoption, highlighting the need for harmonized biosafety and approval protocols.

### 6.4 Future perspectives

To date, only a limited number of plant–microbe interactions have been examined in detail, primarily in a few terrestrial plant species models. Future research encompassing a broader range of plant diversity may reveal novel forms of symbiosis and pathogenesis, including ancient interactions. Biological fertilizers, biopesticides, and microbiome-assisted crop management demonstrate strong potential to enhance agroecosystem resilience.

However, critical future needs include long-term field trials, assessment of climate-driven microbiome shifts, and evaluation of microbiome performance across different soil types and farming systems. Bridging the gap between controlled experiments and real-world agroecosystems remains the most significant challenge for microbiome-based crop protection.

In conclusion, the utilization of microbiomes offers benefits such as improved environmental safety, reduced reliance on chemical pesticides, and enhanced quality of agricultural products. Nevertheless, challenges remain, including the need to develop effective and stable microbial consortia and the difficulties inherent in controlling their effects on plants under diverse agronomic conditions.

## References

[B1] Abadi V. A. J. M. Sepehri M. Rahmani H. A. Zarei M. Ronaghi A. Taghavi S. M. . (2020). Role of dominant phyllosphere bacteria with plant growth–promoting characteristics on growth and nutrition of maize (Zea mays L.). J. Soil Sci. Plant Nutr. 20, 2348–2363. doi: 10.1007/s42729-020-00302-1, PMID: 41758449

[B2] Agbavor C. Mirza B. S. Wait A. (2022). The effects of phyllosphere bacteria on plant physiology and growth of soybean infected with pseudomonas syringae. Plants 11, 2634. doi: 10.3390/plants11192634, PMID: 36235499 PMC9571934

[B3] Akhtar H. Usman M. Binyamin R. Hameed A. Arshad S. F. Aslam H. M. U. . (2024). Traditional strategies and cutting-edge technologies used for plant disease management: A comprehensive overview. Agronomy 14, 2175. doi: 10.3390/agronomy14092175, PMID: 41725453

[B4] Akter T. Maqsood H. Castilla N. Song W. Chen S. (2025). Systems biology applications in revealing plant defense mechanisms in disease triangle. Int. J. Mol. Sci. 26, 7318. doi: 10.3390/ijms26157318, PMID: 40806450 PMC12347815

[B5] Ali S. Tyagi A. Bae H. (2023). Plant microbiome: an ocean of possibilities for improving disease resistance in plants. Microorganisms 11, 392. doi: 10.3390/microorganisms11020392, PMID: 36838356 PMC9961739

[B6] Arıkan M. Muth T. (2023). Integrated multi-omics analyses of microbial communities: A review of the current state and future directions. Mol. Omics. 19, 607–623. doi: 10.1039/D3MO00089C, PMID: 37417894

[B7] Armes A. C. Schaefer A. L. Hochanadel L. H. Klingeman D. M. Carper D. L. Abraham P. E. . (2025). Quorum sensing modulates microbial community structure through regulation of secondary metabolites. mSphere 10, e0105024. doi: 10.1128/msphere.01050-24, PMID: 40539822 PMC12306166

[B8] Arnault G. Marais C. Préveaux A. Briand M. Poisson A. S. Sarniguet A. . (2024). Seedling microbiota engineering using bacterial synthetic community inoculation on seeds. FEMS Microbiol. Ecol. 100. doi: 10.1093/femsec/fiae027, PMID: 38503562 PMC10977042

[B9] Ayaz M. Li C.-H. Ali Q. Zhao W. Chi Y.-K. Shafiq M. . (2023). Bacterial and fungal biocontrol agents for plant disease protection: journey from lab to field, current status, challenges, and global perspectives. Molecules 28, 6735. doi: 10.3390/molecules28186735, PMID: 37764510 PMC10537577

[B10] Azarbad H. Junker R. R. (2024). Biological and experimental factors that define the effectiveness of microbial inoculation on plant traits: a meta-analysis. ISME Commun. 4. doi: 10.1093/ismeco/ycae122, PMID: 39507396 PMC11538580

[B11] Babalola O. O. Adedayo A. A. (2023). Endosphere microbial communities and plant nutrient acquisition toward sustainable agriculture. Emerg. Top. Life Sci. 7, 207–217. doi: 10.1042/ETLS20230069, PMID: 37975608 PMC10754323

[B12] Badri D. V. Chaparro J. M. Zhang R. Shen Q. Vivanco J. M. (2013). Application of natural blends of phytochemicals derived from the root exudates of Arabidopsis to the soil reveal that phenolic-related compounds predominantly modulate the soil microbiome. J. Biol. Chem. 288, 4502–4512. doi: 10.1074/jbc.M112.433300, PMID: 23293028 PMC3576057

[B13] Bakker P. A. Doornbos R. F. Zamioudis C. Berendsen R. L. Pieterse-Bakker C. M. J. . (2013). Induced systemic resistance and the rhizosphere microbiome. Plant Pathol. J. 29, 136–143. doi: 10.5423/PPJ.SI.07.2012.0111, PMID: 25288940 PMC4174772

[B14] Benjamin G. Pandharikar G. Frendo P. (2022). Salicylic acid in plant symbioses: beyond plant pathogen interactions. Biology 11, 861. doi: 10.3390/biology11060861, PMID: 35741382 PMC9220041

[B15] Berg M. Koskella B. (2018). Nutrient- and dose-dependent microbiome-mediated protection against a plant pathogen. Curr. Biol. 28, 2487–2492. doi: 10.1016/j.cub.2018.05.085, PMID: 30057302

[B16] Besserer A. Puech-Pagès V. Kiefer P. Gomez-Roldan V. Jauneau A. Roy S. . (2006). Strigolactones stimulate arbuscular mycorrhizal fungi by activating mitochondria. PLoS Biol. 4, e226. doi: 10.1371/journal.pbio.0040226, PMID: 16787107 PMC1481526

[B17] Besset-Manzoni Y. Joly P. Brutel A. Gerin F. Soudière O. Langin T. . (2019). Does *in vitro* selection of biocontrol agents guarantee success in planta? A study case of wheat protection against Fusarium seedling blight by soil bacteria. PLoS One 14, e0225655. doi: 10.1371/journal.pone.0225655, PMID: 31805068 PMC6894788

[B18] Beye M. Fahsi N. Raoult D. Fournier P. E. (2018). Careful use of 16S rRNA gene sequence similarity values for the identification of Mycobacterium species. New Microbes New Infect. 22, 24–29. doi: 10.1016/j.nmni.2017.12.009, PMID: 29556405 PMC5857167

[B19] Bodhankar S. Grover M. Hemanth S. Reddy G. Rasul S. Yadav S. K. . (2017). Maize seed endophytic bacteria: dominance of antagonistic, lytic enzyme-producing Bacillus spp. 3 Biotech. 7, 232. doi: 10.1007/s13205-017-0860-0, PMID: 28688037 PMC5500752

[B20] Cameron D. D. Neal A. L. Van Wees S. C. M. Ton J. (2013). Mycorrhiza-induced resistance: More than the sum of its parts? Trends Plant Sci. 18, 539–545. doi: 10.1016/j.tplants.2013.06.004, PMID: 23871659 PMC4194313

[B21] Canarini A. Kaiser C. Merchant A. Richter A. Wanek W. (2019). Root exudation of primary metabolites: mechanisms and their roles in plant responses to environmental stimuli. Front. Plant Sci. 10. doi: 10.3389/fpls.2019.00157, PMID: 30881364 PMC6407669

[B22] Caruso D. J. Palombo E. A. Moulton S. E. Zaferanloo B. (2022). Exploring the promise of endophytic fungi: A review of novel antimicrobial compounds. Microorganisms 10, 1990. doi: 10.3390/microorganisms10101990, PMID: 36296265 PMC9607381

[B23] Chao H. Qing Z. Yalan C. Caixia C. Wenquan W. Junling H. . (2021). Colonization by dark septate endophytes improves the growth and rhizosphere soil microbiome of licorice plants under different water treatments. Appl. Soil Ecol. 166. doi: 10.1016/j.apsoil.2021.103993, PMID: 41760527

[B24] Chen Y. Batra H. Dong J. Chen Rao V. B. Tao P. (2019). Genetic engineering of bacteriophages against infectious diseases. Front. Microbiol. 10. doi: 10.3389/fmicb.2019.00954, PMID: 31130936 PMC6509161

[B25] Chen Y. Wang J. Yang N. Wen Z. Sun X. Chai Y. . (2018). Wheat microbiome bacteria can reduce virulence of a plant pathogenic fungus by altering histone acetylation. Nat. Commun. 9, 3429. doi: 10.1038/s41467-018-05683-7, PMID: 30143616 PMC6109063

[B26] Chock M. K. Hoyt B. K. Amend A. S. (2021). Mycobiome transplant increases resistance to Austropuccinia psidii in an endangered Hawaiian plant. Phytobiomes J. 5, 326–334. doi: 10.1094/PBIOMES-09-20-0065-R, PMID: 40211709

[B27] Compant S. Samad A. Faist H. Sessitsch A. (2019). A review on the plant microbiome: Ecology, functions, and emerging trends in microbial application. J. advanced Res. 19, 29–37. doi: 10.1016/j.jare.2019.03.004, PMID: 31341667 PMC6630030

[B28] Copeland C. Schulze-Lefert P. Ma K. W. (2025). Potential and challenges for application of microbiomes in agriculture. Plant Cell 37, koaf185. doi: 10.1093/plcell/koaf185, PMID: 40718998 PMC12379898

[B29] Darbon G. Declerck S. Riot G. Doubell M. Dupuis B. (2024). Inoculation and tracking of beneficial microbes reveal they can establish in field-grown potato roots and decrease blemish diseases. Biol. Fertil Soils 60, 699–712. doi: 10.1007/s00374-024-01822-z, PMID: 41758449

[B30] Dastogeer K. M. G. Tumpa F. H. Sultana A. Akter M. A. Chakraborty A. (2020). Plant microbiome–an account of the factors that shape community composition and diversity. Current Plant Biology 23. doi: 10.1016/j.cpb.2020.100161, PMID: 41760527

[B31] Deb C. R. Tatung M. (2024). Siderophore producing bacteria as biocontrol agent against phytopathogens for a better environment: A review. S Afr J. Bot. 165, 153–162. doi: 10.1016/j.sajb.2023.12.031, PMID: 41760527

[B32] Dhar Purkayastha G. Mangar P. Saha A. Saha D. (2018). Evaluation of the biocontrol efficacy of a Serratia marcescens strain indigenous to tea rhizosphere for the management of root rot disease in tea. PLoS One 13, e0191761. doi: 10.1371/journal.pone.0191761, PMID: 29466418 PMC5821441

[B33] Du Y. Han X. Tsuda K. (2025). Microbiome-mediated plant disease resistance: recent advances and future directions. J. Gen. Plant Pathol. 91, 1–17. doi: 10.1007/s10327-024-01204-1, PMID: 41758449

[B34] Durán P. Thiergart T. Garrido-Oter R. Agler M. Kemen E. Schulze-Lefert P. . (2018). Microbial interkingdom interactions in roots promote arabidopsis survival. Cell 175, 973–983. doi: 10.1016/j.cell.2018.10.020, PMID: 30388454 PMC6218654

[B35] Elamathi E. Malathi P. Viswanathan R. Sundar A. R. (2018). Expression analysis on mycoparasitism related genes during antagonism of Trichoderma with Colletotrichum falcatum causing red rot in sugarcane. J. Plant Biochem. Biotechnol. 27, 351–361. doi: 10.1007/s13562-018-0444-z, PMID: 41758449

[B36] Elhaissoufi W. Ghoulam C. Barakat A. Zeroual Y. Bargaz A. (2021). Phosphate bacterial solubilization: A key rhizosphere driving force enabling higher P use efficiency and crop productivity. J. advanced Res. 38, 13–28. doi: 10.1016/j.jare.2021.08.014, PMID: 35572398 PMC9091742

[B37] Elsayed T. R. Jacquiod S. Nour E. H. Sørensen S. J. Smalla K. (2020). Biocontrol of bacterial wilt disease through complex interaction between tomato plant, antagonists, the indigenous rhizosphere microbiota, and ralstonia solanacearum. Front. Microbiol. 10. doi: 10.3389/fmicb.2019.02835, PMID: 31998244 PMC6967407

[B38] Enebe M. C. Babalola O. O. (2019). The impact of microbes in the orchestration of plants’ resistance to biotic stress: a disease management approach. Appl. Microbiol. Biotechnol. 103, 9–25. doi: 10.1007/s00253-018-9433-3, PMID: 30315353 PMC6311197

[B39] Ezrari S. Mhidra O. Radouane N. Tahiri A. Polizzi G. Lazraq A. . (2021). Potential role of rhizobacteria isolated from citrus rhizosphere for biological control of citrus dry root rot. Plants 10, 872. doi: 10.3390/plants10050872, PMID: 33926049 PMC8145030

[B40] Fadiji A. E. Babalola O. O. (2020). Exploring the potentialities of beneficial endophytes for improved plant growth. Saudi J. Biol. Sci. 27, 3622–3633. doi: 10.1016/j.sjbs.2020.08.002, PMID: 33304173 PMC7714962

[B41] Ferrarini E. Špacapan M. Lam V. B. Mccann A. Cesa-Luna C. Marahatta B. P. . (2022). Versatile role of Pseudomonas fuscovaginae cyclic lipopeptides in plant and microbial interactions. Front. Plant Sci. 13. doi: 10.3389/fpls.2022.1008980, PMID: 36426159 PMC9679282

[B42] Flemer B. Gulati S. Bergna A. Rändler M. Cernava T. Witzel K. . (2022). Biotic and abiotic stress factors induce microbiome shifts and enrichment of distinct beneficial bacteria in tomato roots. Phytobiomes J. 6, 276–289. doi: 10.1094/PBIOMES-10-21-0067, PMID: 40211709

[B43] Foo J. L. Ling H. Lee Y. S. Chang M. W. (2017). Microbiome engineering: Current applications and its future. Biotechnol. J. 12. doi: 10.1002/biot.201600099, PMID: 28133942

[B44] Glick B. R. Gamalero E. (2021). Recent developments in the study of plant microbiomes. Microorganisms 9, 1533. doi: 10.3390/microorganisms9071533, PMID: 34361969 PMC8306116

[B45] Gross S. Kunz L. Müller D. C. Santos Kron A. Freimoser F. M. (2018). Characterization of antagonistic yeasts for biocontrol applications on apples or in soil by quantitative analyses of synthetic yeast communities. Yeast (Chichester England) 35, 559–566. doi: 10.1002/yea.3321, PMID: 29752875 PMC6220783

[B46] Gu S. Wei Z. Shao Z. Friman V. P. Cao K. Yang T. . (2020). Competition for iron drives phytopathogen control by natural rhizosphere microbiomes. Nat. Microbiol. 5, 1002–1010. doi: 10.1038/s41564-020-0719-8, PMID: 32393858 PMC7116525

[B47] Gu Y. Wei Z. Wang X. Friman V. P. Huang J. Wang X. . (2016). Pathogen invasion indirectly changes the composition of soil microbiome via shifts in root exudation profile. Biol. Fertil Soils 52, 997–1005. doi: 10.1007/s00374-016-1136-2, PMID: 41758449

[B48] Guo Q. Li Y. Lou Y. Shi M. Jiang Y. Zhou J. . (2019). Bacillus amyloliquefaciens Ba13 induces plant systemic resistance and improves rhizosphere microecology against tomato yellow leaf curl virus disease. Appl. Soil Ecol. 137, 154–166. doi: 10.1016/j.apsoil.2019.01.015, PMID: 41760527

[B49] Gupta R. Anand G. Gaur R. Et al. (2021). Plant–microbiome interactions for sustainable agriculture: a review. Physiol. Mol. Biol. Plants 27, 165–179. doi: 10.1007/s12298-021-00927-1, PMID: 33627969 PMC7873154

[B50] Guzmán-Guzmán P. Etesami H. Santoyo G. (2025). Trichoderma: a multifunctional agent in plant health and microbiome interactions. BMC Microbiol. 25, 434. doi: 10.1186/s12866-025-04158-2, PMID: 40652165 PMC12255041

[B51] Hamaoka K. Aoki Y. Suzuki S. (2021). Isolation and characterization of endophyte bacillus velezensis KOF112 from grapevine shoot xylem as biological control agent for fungal diseases. Plants 10, 1815. doi: 10.3390/plants10091815, PMID: 34579349 PMC8468208

[B52] Huang S. Zha X. Fu G. (2023). Affecting factors of plant phyllosphere microbial community and their responses to climatic warming—A review. Plants 12, 2891. doi: 10.3390/plants12162891, PMID: 37631103 PMC10458011

[B53] Innerebner G. Knief C. Vorholt J. A. (2011). Protection of Arabidopsis thaliana against leaf-pathogenic Pseudomonas syringae by Sphingomonas strains in a controlled model system. Appl. Environ. Microbiol. 77, 3202–3210. doi: 10.1128/AEM.00133-11, PMID: 21421777 PMC3126462

[B54] Islam W. Noman A. Naveed H. Huang Z. Chen H. Y. H. (2020). Role of environmental factors in shaping the soil microbiome. Environ. Sci. pollut. Res. Int. 27, 41225–41247. doi: 10.1007/s11356-020-10471-2, PMID: 32829437

[B55] Jabran M. Ali M. A. Acet T. Zahoor A. Abbas A. Arshad U. . (2024). Growth regulation in bread wheat via novel bioinoculant formulation. BMC Plant Biol. 24, 1039. doi: 10.1186/s12870-024-05698-x, PMID: 39491015 PMC11533284

[B56] Jabran M. Ali M. A. Zahoor A. Muhae-Ud-Din G. Liu T. Chen W. . (2023). Intelligent reprogramming of wheat for enhancement of fungal and nematode disease resistance using advanced molecular techniques. Front. Plant Sci. 14. doi: 10.3389/fpls.2023.1132699, PMID: 37235011 PMC10206142

[B57] Jacoby R. P. Chen L. Schwier M. Koprivova A. Kopriva S. (2020). Recent advances in the role of plant metabolites in shaping the root microbiome. F1000Research 9, F1000 Faculty Rev–151. doi: 10.12688/f1000research.21796.1, PMID: 32148778 PMC7047909

[B58] Jaiswal D. K. Gawande S. J. Soumia P. S. Krishna R. Vaishnav A. Ade A. B. . (2022). Biocontrol strategies: an eco-smart tool for integrated pest and diseases management. BMC Microbiol. 22, 324. doi: 10.1186/s12866-022-02744-2, PMID: 36581846 PMC9801620

[B59] Jing J. Garbeva P. Raaijmakers J. M. Medema M. H. (2024). Strategies for tailoring functional microbial synthetic communities. ISME J. 18, 244–261. doi: 10.1093/ismejo/wrae049, PMID: 38537571 PMC11008692

[B60] Jog R. Nareshkumar G. Rajkumar S. (2016). “ Enhancing soil health and plant growth promotion by actinomycetes,” in Plant growth promoting actinobacteria ( Springer Nature, Berlin), 33–45. doi: 10.1007/978-981-10-0707-1_3, PMID:

[B61] Jones D. L. Magthab E. A. Gleeson D. B. Hill P. W. Sánchez-Rodríguez A. R. Roberts P. . (2018). Microbial competition for nitrogen and carbon is as intense in the subsoil as in the topsoil. Soil Biol. Biochem. 117, 72–82. doi: 10.1016/j.soilbio.2017.10.024, PMID: 41760527

[B62] Jumpponen A. Mattson K. G. Trappe J. M. (1998). Mycorrhizal functioning of Phialocephala fortinii with Pinus contorta on glacier forefront soil: Interactions with soil nitrogen and organic matter. Mycorrhiza 7, 261–265. doi: 10.1007/s005720050190, PMID: 24578052

[B63] Kageyama S. A. Mandyam K. G. Jumpponen A. (2008). “ Diversity, functionand potential applications of the root-associated endophytes,” in Mycorrhiza. Ed. Varma A. ( Springer, Berlin), 29–57. doi: 10.1007/978-3-540-78826-3_2, PMID:

[B64] Ke J. Wang B. Yoshikuni Y. (2021). Microbiome engineering: synthetic biology of plant-associated microbiomes in sustainable agriculture. Trends Biotechnol. 39, 244–261. doi: 10.1016/j.tibtech.2020.07.008, PMID: 32800605

[B65] Khadieva G. F. Lutfullin M. T. Mochalova N. K. Mardanova A. M. Sharipova M. R. (2018). Probiotic based on bacterial strain Bacillus subtilis MG-8 VKPM V-12476 and method of application probiotic for prevention of gastrointestinal diseases in agricultural animals and poultryRU 2, 663, 720 C1 (Kazan: The Federal Service for Intellectual Property).

[B66] Kim S. H. Vujanovic V. (2017). Biodegradation and biodetoxification of Fusarium mycotoxins by Sphaerodes mycoparasitica. AMB Expr 7, 145. doi: 10.1186/s13568-017-0446-6, PMID: 28687037 PMC5500597

[B67] Kim S. H. Vujanovic V. (2022). ATP-Binding Cassette (ABC) Transporters in Fusarium Specific Mycoparasite Sphaerodes mycoparasitica during Biotrophic Mycoparasitism. Appl. Sci. 12, 7641. doi: 10.3390/app12157641, PMID: 41725453

[B68] Kimotho R. N. Maina S. (2024). Unraveling plant-microbe interactions: can integrated omics approaches offer concrete answers? J. Exp. Bot. 75, 1289–1313. doi: 10.1093/jxb/erad448, PMID: 37950741 PMC10901211

[B69] Koike N. Hyakumachi M. Kageyama K. Tsuyumu S. Doke-Koike N. (2001). Induction of Systemic Resistance in Cucumber against Several Diseases by Plant Growth-promoting Fungi: lignification and Superoxide Generation. Eur. J. Plant Pathol. 107, 523–533. doi: 10.1023/A:1011203826805, PMID: 40797221

[B70] Kong Z. Hart M. Liu H. (2018). Paving the way from the lab to the field: Using synthetic microbial consortia to produce high-quality crops. In Front. Plant Sci. 9. doi: 10.3389/fpls.2018.01467, PMID: 30344529 PMC6182091

[B71] Kour D. Negi R. Khan S. S. Kumar S. Kaur S. Kaur T. . (2024). Microbes mediated induced systemic response in plants: a review. Plant Stress. 11, 100334. doi: 10.1016/j.stress.2023.100334, PMID: 41760527

[B72] Kowalska J. Krzymińska J. Tyburski J. (2022). Yeasts as a potential biological agent in plant disease protection and yield improvement—A short review. Agriculture 12, 1404. doi: 10.3390/agriculture12091404, PMID: 41725453

[B73] Lahlali R. Ezrari S. Radouane N. Kenfaoui J. Esmaeel Q. El Hamss H. . (2022). Biological control of plant pathogens: A global perspective. Microorganisms 10, 596. doi: 10.3390/microorganisms10030596, PMID: 35336171 PMC8951280

[B74] Ledermann R. Schulte C. C. M. Poole P. S. (2021). How rhizobia adapt to the nodule environment. J. bacteriology 203, e0053920. doi: 10.1128/JB.00539-20, PMID: 33526611 PMC8315917

[B75] Ledru L. Garnier J. Rhor M. Nous C. Ibanez S. -L. (2022). Mutualists construct the ecological conditions that trigger the transition from parasitism. Peer Community 2, e41. doi: 10.24072/pcjournal.139, PMID: 41369854

[B76] Legein M. Smets W. Vandenheuvel D. Eilers T. Muyshondt B. Prinsen E. . (2020). Modes of action of microbial biocontrol in the phyllosphere. Front. Microbiol. 11. doi: 10.3389/fmicb.2020.01619, PMID: 32760378 PMC7372246

[B77] Lelyak A. I. Lelyak A. A. (2013). Strains of Bacillus subtilis and Bacillus amyloliquefaciens bacteria promoting recovery in soil and animal gastrointestinal tracts, possessing bactericidal, fungicidal and virucidal activity and preparation in basis of same strains.RU 2,482,174 C2 (Moscow: The Federal Service for Intellectual Property).

[B78] Le Mire G. Siah A. Brisset M.-N. Gaucher M. Deleu M. Jijakli M. H. (2018). Surfactin Protects Wheat against Zymoseptoria tritici and Activates Both Salicylic Acid- and Jasmonic Acid-Dependent Defense Responses. Agriculture 8, 11. doi: 10.3390/agriculture8010011, PMID: 41725453

[B79] Li Z. Bai X. Jiao S. Li Y. Li P. Yang Y. . (2021). A simplified synthetic community rescues Astragalus mongholicus from root rot disease by activating plant-induced systemic resistance. Microbiome 9, 217. doi: 10.1186/s40168-021-01169-9, PMID: 34732249 PMC8567675

[B80] Li X. He X. Hou L. Ren Y. Wang S. Su F. (2018). Dark septate endophytes isolated from a xerophyte plant promote the growth of Ammopiptanthus mongolicus under drought condition. Sci. Rep. 8, 7896. doi: 10.1038/s41598-018-26183-0, PMID: 29785041 PMC5962579

[B81] Li Q. Hou Z. Zhou D. Jia M. Lu S. Yu J. (2022). A plant growth-promoting bacteria Priestia megaterium JR48 induces plant resistance to the crucifer black rot via a Salicylic acid-dependent signaling pathway. Front. Plant Sci. 13. doi: 10.3389/fpls.2022.1046181, PMID: 36438094 PMC9684715

[B82] Li P. D. Zhu Z. R. Zhang Y. Xu J. Wang H. Wang Z. . (2022). The phyllosphere microbiome shifts toward combating melanose pathogen. Microbiome 10, 56. doi: 10.1186/s40168-022-01234-x, PMID: 35366955 PMC8976405

[B83] Liotti R. G. Da Silva Figueiredo M. I. Da Silva G. F. De Mendonça E. A. F. Soares M. A. (2018). Diversity of cultivable bacterial endophytes in Paullinia cupana and their potential for plant growth promotion and phytopathogen control. Microbiological Res. 207, 8–18. doi: 10.1016/j.micres.2017.10.011, PMID: 29458872

[B84] Liu P. Luo L. Long C. A. (2013). Characterization of competition for nutrients in the biocontrol of Penicillium italicum by Kloeckeraapiculata. Biol. Control 67, 157–162. doi: 10.1016/j.biocontrol.2013.07.011, PMID: 41760527

[B85] Liu Y. Yan H. Zhang X. Zhang R. Li M. Xu T. . (2020). Investigating the endophytic bacterial diversity and community structures in seeds of genetically related maize (Zea mays L.) genotypes. 3 Biotech. 10, 27. doi: 10.1007/s13205-019-2034-8, PMID: 31950006 PMC6942555

[B86] Liu Y. Zhu A. Tan H. Cao L. Zhang R. (2019). Engineering banana endosphere microbiome to improve Fusarium wilt resistance in banana. Microbiome 7, 74. doi: 10.1186/s40168-019-0690-x, PMID: 31092296 PMC6521393

[B87] Liu Y. Zuo S. Xu L. Zou Y. Song W. (2012). Study on diversity of endophytic bacterial communities in seeds of hybrid maize and their parental lines. Arch. Microbiol. 194, 1001–1012. doi: 10.1007/s00203-012-0836-8, PMID: 22892578

[B88] Loreti E. Perata P. (2020). The many facets of hypoxia in plants. Plants (Basel Switzerland) 9, 745. doi: 10.3390/plants9060745, PMID: 32545707 PMC7356549

[B89] Lü P. Liu Y. Yu X. Shi C. L. Liu X. (2022). The right microbe-associated molecular patterns for effective recognition by plants. Front. Microbiol. 13. doi: 10.3389/fmicb.2022.1019069, PMID: 36225366 PMC9549324

[B90] Lyu X. Nuhu M. Candry P. Wolfanger J. Betenbaugh M. Saldivar A. . (2024). Top-down and bottom-up microbiome engineering approaches to enable biomanufacturing from waste biomass. J. Ind. Microbiol. Biotechnol. 51, kuae025. doi: 10.1093/jimb/kuae025, PMID: 39003244 PMC11287213

[B91] Maachia B. Rafik E. Chérif M. Nandal P. Mohapatra T. Bernard P. (2015). Biological control of the grapevine diseases ‘grey mold’ and ‘powdery mildew’ by Bacillus B27 and B29 strains. Indian J. Exp. Biol. 53, 109–115. 25757242

[B92] Maclean A. M. Bravo A. Harrison M. J. (2017). Plant signaling and metabolic pathways enabling arbuscular mycorrhizal symbiosis. Plant Cell 29, 2319–2335. doi: 10.1105/tpc.17.00555, PMID: 28855333 PMC5940448

[B93] Mandyam K. Jumpponen A. (2005). Seeking the elusive function of the root-colonising dark septate endophytic fungi. Stud. Mycol. 53, 173–189. doi: 10.3114/sim.53.1, PMID: 41551220

[B94] Mandyam K. G. Jumpponen A. (2015). Mutualism–parasitism paradigm synthesized from results of root-endophyte models. Front. Microbiol. 5. doi: 10.3389/fmicb.2014.00776, PMID: 25628615 PMC4290590

[B95] Marín O. González B. Poupin M. J. (2021). From microbial dynamics to functionality in the rhizosphere: A systematic review of the opportunities with synthetic microbial communities. Front. Plant Sci. 12. doi: 10.3389/fpls.2021.650609, PMID: 34149752 PMC8210828

[B96] Meena M. Swapnil P. Divyanshu K. Kumar S. Harish, Tripathi Y. N. Zehra A. . (2020). PGPR-mediated induction of systemic resistance and physiochemical alterations in plants against the pathogens: Current perspectives. J. basic Microbiol. 60, 828–861. doi: 10.1002/jobm.202000370, PMID: 32815221

[B97] Mendes R. Garbeva P. Raaijmakers J. M. (2013). The rhizosphere microbiome: significance of plant beneficial, plant pathogenic, and human pathogenic microorganisms. FEMS Microbiol. Rev. 37, 634–663. doi: 10.1111/1574-6976.12028, PMID: 23790204

[B98] Miller S. A. Ferreira J. P. Lejeune J. T. (2022). Antimicrobial use and resistance in plant agriculture: A one health perspective. Agriculture 12, 289. doi: 10.3390/agriculture12020289, PMID: 41725453

[B99] Moënne-Loccoz Y. Mavingui P. Combes C. Normand P. Steinberg C. (2014). Microorganisms and biotic interactions. Environmental microbiology: fundamentals and applications: microbial ecology (Dordrecht: Springer), 395–444. doi: 10.1007/978-94-017-9118-2_11, PMID:

[B100] Molina L. Constantinescu F. Michel L. Reimmann C. Duffy B. Défago G. (2003). Degradation of pathogen quorum-sensing molecules by soil bacteria: a preventive and curative biological control mechanism. FEMS Microbiol. Ecol. 45, 71–81. doi: 10.1016/S0168-6496(03)00125-9, PMID: 19719608

[B101] Morella N. M. Zhang X. Koskella B. (2019). Tomato seed-associated bacteria confer protection of seedlings against foliar disease caused by Pseudomonas syringae. Phytobiomes J. 3, 177–190. doi: 10.1094/PBIOMES-01-19-0007-R, PMID: 40211709

[B102] Netherway T. Bengtsson J. Buegger F. Fritscher J. Oja J. Pritsch K. . (2024). Pervasive associations between dark septate endophytic fungi with tree root and soil microbiomes across Europe. Nat. Commun. 15, 159. doi: 10.1038/s41467-023-44172-4, PMID: 38167673 PMC10761831

[B103] Ning J. Gang G. Bai Z. Hu Q. Qi H. Ma A. . (2012). *In situ* enhanced bioremediation of dichlorvos by a phyllosphere Flavobacterium strain. Front. Environ. Sci. Eng. 6, 231–237. doi: 10.1007/s11783-011-0316-4, PMID: 41758449

[B104] Niu B. Paulson J. N. Zheng. X. Kolter R. (2017). Simplified and rep-resentative bacterial community of maize roots. Proc. Natl. Acad. Sci. 114, 2450–2459. doi: 10.1073/pnas.1616148114, PMID: 28275097 PMC5373366

[B105] Pandit M. A. Kumar J. Gulati S. Bhandari N. Mehta P. Katyal R. . (2022). Major biological control strategies for plant pathogens. Pathogens 11, 273. doi: 10.3390/pathogens11020273, PMID: 35215215 PMC8879208

[B106] Pantigoso H. A. Newberger D. Vivanco J. M. (2022). The rhizosphere microbiome: Plant-microbial interactions for resource acquisition. J. Appl. Microbiol. 133, 2864–2876. doi: 10.1111/jam.15686, PMID: 36648151 PMC9796772

[B107] Peterson S. B. Dunn A. K. Klimowicz A. K. Handelsman J. (2006). Peptidoglycan from Bacillus cereus mediates commensalism with rhizosphere bacteria from the Cytophaga-Flavobacterium group. Appl. Environ. Microbiol. 72, 5421–5427. doi: 10.1128/AEM.02928-05, PMID: 16885294 PMC1538759

[B108] Pradhan S. Tyagi S. Sharma S. (2022). Combating biotic stresses in plants by synthetic microbial communities: Principles, applications and challenges. J. Appl. Microbiol. 133, 2742–2759. doi: 10.1111/jam.15799, PMID: 36039728

[B109] Prajapati S. Kumar N. Kumar S. Lakhran L. Maurya S. (2020). Biological control a sustainable approach for plant diseases management: A review. J. Pharmacognosy Phytochem. 9, 1514–1523.

[B110] Rolfe S. A. Griffiths J. Ton J. (2019). Crying out for help with root exudates: adaptive mechanisms by which stressed plants assemble health-promoting soil microbiomes. Curr. Opin. Microbiol. 49, 73–82. doi: 10.1016/j.mib.2019.10.003, PMID: 31731229

[B111] Romero F. M. Rossi F. R. Gárriz A. Carrasco P. Ruíz O. A. (2019). A bacterial endophyte from apoplast fluids protects canola plants from different phytopathogens via antibiosis and induction of host resistance. Phytopathology 109, 375–383. doi: 10.1094/PHYTO-07-18-0262-R, PMID: 30156501

[B112] Russ D. Fitzpatrick C. R. Teixeira P. J. P. L. Dangl J. L. (2023). Deep discovery informs difficult deployment in plant microbiome science. In Cell 18, 4496–4513. doi: 10.1016/j.cell.2023.08.035, PMID: 37832524

[B113] Saeed Q. Xiukang W. Haider F. U. Kuˇcerik J. Mumtaz M. Z. Holatko J. . (2021). Rhizosphere bacteria in plant growth promotion, biocontrol, and bioremediation of contaminated sites: A comprehensive review of effects and mechanisms. Int. J. Mol. Sci. 22, 10529. doi: 10.3390/ijms221910529, PMID: 34638870 PMC8509026

[B114] Santhanam R. Luu V. T. Weinhold A. Goldberg J. Oh Y. Baldwin I. T. (2015). Native root-associated bacteria rescue a plant from a sudden-wilt disease that emerged during continuous cropping. Proc. Natl. Acad. Sci. United States America 112, 5013–5020. doi: 10.1073/pnas.1505765112, PMID: 26305938 PMC4568709

[B115] Santos M. Cesanelli I. Diánez F. Sánchez-Montesinos B. Moreno-Gavíra A. (2021). Advances in the role of dark septate endophytes in the plant resistance to abiotic and biotic stresses. J. Fungi 7, 939. doi: 10.3390/jof7110939, PMID: 34829226 PMC8622582

[B116] Santos L. Olivares F. (2021). Plant microbiome structure and benefits for sustainable agriculture. Curr. Plant Biol. 26, 100198. doi: 10.1016/j.cpb.2021.100198, PMID: 41760527

[B117] Santoyo G. (2022). How plants recruit their microbiome? New insights into beneficial interactions. J. advanced Res. 40, 45–58. doi: 10.1016/j.jare.2021.11.020, PMID: 36100333 PMC9481936

[B118] Schalk I. J. (2008). Metal trafficking via siderophores in Gram-negative bacteria: specificities and characteristics of the pyoverdine pathway. J. inorganic Biochem. 102, 1159–1169. doi: 10.1016/j.jinorgbio.2007.11.017, PMID: 18221784

[B119] Schmidt C. S. Mrnka L. Lovecká P. Frantík T. Fenclová M. Demnerová K. . (2021). Bacterial and fungal endophyte communities in healthy and diseased oilseed rape and their potential for biocontrol of Sclerotinia and Phoma disease. Sci. Rep. 11, 3810. doi: 10.1038/s41598-021-81937-7, PMID: 33589671 PMC7884388

[B120] Shameer S. Prasad T. (2018). Plant growth promoting rhizobacteria for sustainable agricultural practices with special reference to biotic and abiotic stresses. Plant Growth Regul. 84, 603–615. doi: 10.1007/s10725-017-0365-1, PMID: 41758449

[B121] Shao Z. Gu S. Zhang X. Xue J. Yan T. Guo S. . (2024). Siderophore interactions drive the ability of Pseudomonas spp. consortia to protect tomato against Ralstonia solanacearum. Horticulture Res. 11. doi: 10.1093/hr/uhae186, PMID: 39247881 PMC11377186

[B122] Shao Z. Schenk P. M. Dart P. (2023). Phyllosphere bacterial strains Rhizobium b1 and Bacillus subtilis b2 control tomato leaf diseases caused by Pseudomonas syringae pv. tomato and Alternaria solani. J. Appl. Microbiol. 134. doi: 10.1093/jambio/lxad139, PMID: 37422439

[B123] Sharma A. Shahzad V. K. B. Tanveer M. Sidhu G. P. S. Handa N. Kohli S. K. . (2019). Worldwide pesticide usage and its impacts on ecosystem. SN Appl. Sci. 1, 1446. doi: 10.1007/s42452-019-1485-1, PMID: 41758449

[B124] Sharma R. Sindhu S. Sindhu S. S. (2018). Suppression of alternaria blight disease and plant growth promotion of mustard (BrassicaJuncea L.) by antagonistic rhizosphere bacteria. Appl. Soil Ecol. 129, 145–150. doi: 10.1016/j.apsoil.2018.05.013, PMID: 41760527

[B125] Shayanthan A. Ordoñez P. A. C. Oresnik I. J. (2022). The role of synthetic microbial communities (SynCom) in sustainable agriculture. Front. Agron. 4. doi: 10.3389/fagro.2022.896307, PMID: 41757362

[B126] Smith S. E. Read D. J. (2002). Arbutoid and monotropoid mycorrhizas. Mycorrhizal Symbiosis 2, 301–322. doi: 10.1016/b978-012652840-4/50012-7, PMID: 41760527

[B127] Sobanbabu G. Oviya R. Meena B. Vijayasamundeeswari A. Shanmugaiah V. Ramamoorthy V. (2024). Evaluation of phyllosphere bacterial biocontrol agents for the suppression of rice foliar diseases. J. Phytopathol. 172, e1330 0. doi: 10.1111/jph.13300, PMID: 41744481

[B128] Song W. Shao H. Zheng A. Zhao L. Xu Y. (2023). Advances in roles of salicylic acid in plant tolerance responses to biotic and abiotic stresses. Plants 12, 3475. doi: 10.3390/plants12193475, PMID: 37836215 PMC10574961

[B129] Sun H. Jiang S. Jiang C. Wu C. Gao M. Wang Q. (2021). A review of root exudates and rhizosphere microbiome for crop production. Environ. Sci. pollut. Res. Int. 28, 54497–54510. doi: 10.1007/s11356-021-15838-7, PMID: 34431053

[B130] Tanaka E. Umeki D. Kido S. Makishima R. Tamaki Y. Murakami T. . (2025). Biocontrol of bacterial wilt disease using plant-associated bacterial communities in tomato. Mol. Plant-Microbe interactions: MPMI 38, 411–426. doi: 10.1094/MPMI-09-24-0114-R, PMID: 40354312

[B131] Tao J. Gu M. Yu S. Shi J. Cheng L. Jin J. . (2024). The beneficial endophytic microbes enhanced tobacco defense system to resist bacterial wilt disease. Chem. Biol. Technol. Agric. 11, 21. doi: 10.1186/s40538-024-00542-8, PMID: 41762274

[B132] Tariq A. Guo S. Farhat F. Shen X. (2025). Engineering synthetic microbial communities: diversity and applications in soil for plant resilience. In Agron. 15, 513. doi: 10.3390/agronomy15030513, PMID: 41725453

[B133] Tharanath A. C. Upendra R. S. Rajendra K. (2024). Soil symphony: A comprehensive overview of plant–microbe interactions in agricultural systems. Appl. Microbiol. 4, 1549–1567. doi: 10.3390/applmicrobiol4040106, PMID: 41725453

[B134] Tikhonovich I. A. Provorov N. A. (2003). Simbiogenetics of microbe-plant interactions. Ecol. Genet. 1, 36–46. doi: 10.17816/ecogen1036-46, PMID: 40927655

[B135] Tyagi A. Lama Tamang T. Kashtoh H. Mir R. A. Mir Z. A. Manzoor S. . (2024). A review on biocontrol agents as sustainable approach for crop disease management: applications, production, and future perspectives. Horticulturae 10, 805. doi: 10.3390/horticulturae10080805, PMID: 41725453

[B136] Vandana U. K. Rajkumari J. Singha L. P. Satish L. Alavilli H. Sudheer P. D. V. N. . (2021). The Endophytic Microbiome as a hotspot of Synergistic Interactions, with prospects of Plant Growth Promotion. Biology 10, 101. doi: 10.3390/biology10020101, PMID: 33535706 PMC7912845

[B137] Vinale F. Sivasithamparam K. Ghisalberti E. L. Marra R. Woo S. L. Lorito M. (2008). Trichoderma-plant-pathogen interactions. Soil Biol. Biochem. 40, 1–10. doi: 10.1016/j.soilbio.2007.07.002, PMID: 41760527

[B138] Wang C. Kuzyakov Y. (2024). Mechanisms and implications of bacterial-fungal competition for soil resources. ISME J. 18. doi: 10.1093/ismejo/wrae073, PMID: 38691428 PMC11104273

[B139] Wang D. Liu B. Ma Z. Feng J. Yan H. (2021). Reticine A, a new potent natural elicitor: isolation from the fruit peel of Citrus reticulate and induction of systemic resistance against tobacco mosaic virus and other plant fungal diseases. Pest Manage. Sci. 77, 354–364. doi: 10.1002/ps.6025, PMID: 32741113

[B140] Wang Y. Tian Z. Li Y. Nan Z. Duan T. (2025). Arbuscular mycorrhizal fungus alters the phyllosphere microbial community and modifies plant defences against co-attack of pea aphid and pathogen. Funct. Ecol. 39, 3181–3196. doi: 10.1111/1365-2435.70169, PMID: 41744481

[B141] Wang X. Wang S. Huang M. He Y. Guo S. Yang K. . (2024). Phages enhance both phytopathogen density control and rhizosphere microbiome suppressiveness. MBio 15. doi: 10.1128/mbio.03016-23, PMID: 38780276 PMC11237578

[B142] Wankhade A. Wilkinson E. Britt D. W. Kaundal A. (2025). A review of plant–microbe interactions in the rhizosphere and the role of root exudates in microbiome engineering. Appl. Sci. 15, 7127. doi: 10.3390/app15137127, PMID: 41725453

[B143] Wei Z. Yang X. Yin S. Shen Q. Ran W. Xu Y. (2011). Efficacy of Bacillus-fortified organic fertiliser in controlling bacterial wilt of tomato in the field. Appl. Soil Ecol. 48, 152–159. doi: 10.1016/j.apsoil.2011.03.013, PMID: 41760527

[B144] Xie J. Wang X. Xu J. Xie H. Cai Y. Liu Y. . (2021). Strategies and structure feature of the aboveground and belowground microbial community respond to drought in wild rice (Oryza longistaminata). Rice 14, 79. doi: 10.1186/s12284-021-00522-8, PMID: 34495440 PMC8426455

[B145] Xiong C. Zhu Y. G. Wang J. T. Singh B. Han L. L. Shen J. P. . (2021). Host selection shapes crop microbiome assembly and network complexity. New Phytol. 229, 1091–1104. doi: 10.1111/nph.16890, PMID: 32852792

[B146] Xu X. Dinesen C. Pioppi A. Kovács Á.T. Lozano-Andrade C. N. (2025). Composing a microbial symphony: synthetic communities for promoting plant growth. Trends Microbiol. 33, 738–751. doi: 10.1016/j.tim.2025.01.006, PMID: 39966007

[B147] Yin C. Hagerty C. H. Paulitz T. C. (2022). Synthetic microbial consortia derived from rhizosphere soil protect wheat against a soilborne fungal pathogen. Front. Microbiol. 13. doi: 10.3389/fmicb.2022.908981, PMID: 36118206 PMC9473337

[B148] Yuan Q. S. Gao Y. Wang L. Wang X. Wang L. Ran J. . (2024). Pathogen-driven Pseudomonas reshaped the phyllosphere microbiome in combination with Pseudostellaria heterophylla foliar disease resistance via the release of volatile organic compounds. Environ. Microbiome 19, 61. doi: 10.1186/s40793-024-00603-3, PMID: 39182153 PMC11344943

[B149] Yuan J. Zhao J. Wen T. Zhao M. Li R. Goossens P. . (2018). Root exudates drive the soil-borne legacy of aboveground pathogen infection. Microbiome 6, 156. doi: 10.1186/s40168-018-0537-x, PMID: 30208962 PMC6136170

[B150] Zahoor A. Arshad U. Niaz Z. Shafique M. S. Sarwar N. Jabran M. (2024). Reshaping the plant microbiomes for sustainable disease management. Integr. Plant Biotechnol. 2, 63–75. doi: 10.55627/pbiotech.002.01.0786

[B151] Zehra A. Raytekar N. A. Meena M. Swapnil P. (2021). Efficiency of microbial bio-agents as elicitors in plant defense mechanism under biotic stress: A review. Curr. Res. microbial Sci. 2, 100054. doi: 10.1016/j.crmicr.2021.100054, PMID: 34841345 PMC8610294

[B152] Zhang Y. Jing M. Lyu L. Nie L. Xu X. Sun R. . (2025). Principles for rigorous design and application of synthetic microbial communities. Adv. Sci. doi: 10.1002/advs.202514750, PMID: 41420838 PMC12915082

[B153] Zhu H. Zhou H. Ren Z. Liu E. (2022). Control of Magnaporthe oryzae and Rice Growth Promotion by Bacillus subtilis JN005. J. Plant Growth Regul. 41, 2319–2327. doi: 10.1007/s00344-021-10444-w, PMID: 41758449

